# Effects of simulated multi-sensory stimulation integration on physiological and psychological restoration in virtual urban green space environment

**DOI:** 10.3389/fpsyg.2024.1382143

**Published:** 2024-06-20

**Authors:** Chen Song, Saixin Cao, Hao Luo, Yinghui Huang, Siwei Jiang, Baimeng Guo, Nian Li, Kai Li, Ping Zhang, Chunyan Zhu, Erkang Fu, Mingyan Jiang, Xi Li

**Affiliations:** ^1^College of Landscape Architecture, Sichuan Agricultural University, Chengdu, China; ^2^Guangdong Provincial Key Laboratory of Silviculture, Protection and Utilization, Guangdong Academy of Forestry, Guangzhou, China

**Keywords:** urban green spaces, virtual stimulation, multisensory stimulation, physical restoration, mental restoration

## Abstract

Virtual urban green environment images and audio stimuli had been proven to have restorative effects on subjects’ physical and mental health. In this area, researchers predominantly focused on visual, auditory and olfactory aspects, while tactile and gustatory senses have been minimally explored. However, the optimal combination of sensory stimuli for promoting physical and mental recovery remains unclear. Therefore, a simulated sensory stimulation approach involving 240 participants was employed, with 30 individuals included in each of the eight experimental groups: the visual–auditory (VA), visual–auditory-olfactory (VAO), visual–auditory-tactile (VAT), visual–auditory-gustatory(VAG), visual–auditory-olfactory-tactile (VAOT), visual–auditory-olfactory-gustatory (VAOG), visual–auditory-tactile-gustatory (VATG), and visual–auditory-olfactory-tactile-gustatory (VAOTG) groups. This study aimed to explore the differences in participants’ physiological and psychological health recovery after exposure to different combinations of simulated sensory stimuli in virtual UGSs. The results indicated that the following: (1) In terms of physiological recovery, the blood pressure of the 8 experimental groups decreased significantly after the experiment, indicating that the virtual urban green space environment has a certain recovery effect on physiological state. The combination of VAOTG stimuli in the multisensory group resulted in the best blood pressure recovery (*p* < 0.05). Tactile is an important sense to enhance the physiological recovery effect. Olfactory-tactile or tactile-gustatory stimuli interactions significantly enhance physiological recovery, emphasizing the importance of tactile stimulation in improving physiological recovery. (2) In terms of psychological recovery, the common trigger of olfactory-gustatory is the most key element to enhance psychological recovery through multi-sensory stimulation of virtual urban green space environment. VAOG stimulation had the best effect on psychological recovery (*p* < 0.05), followed by VAOTG stimulation (*p* < 0.05). Gustatory is an important sense to enhance the psychological recovery effect, and both the tactile-gustatory interaction and the olfactory-gustatory interaction significantly enhance the recovery effect. At the same time, the psychological recovery effect obtained by four or more sensory combinations was higher than that obtained by two or three sensory stimulation groups. This study confirms more possibilities for ways to restore physical and mental health through virtual natural environments. It expands the research on the benefits of virtual nature experience and provides theoretical support for the application of this method.

## 1 Introduction

In recent years, numerous studies have shown that UGSs contribute to mitigating health losses among city dwellers by positively influencing emotional well-being (McMahan and Estes, 2015; Pirchio et al., 2021), alleviating anxiety and stress (Hedblom et al., 2019; Pirchio et al., 2021), enhancing cognitive abilities (Bratman et al., 2012), and reducing the incidence and mortality rates of cardiovascular and respiratory diseases (Maas et al., 2006; Tsunetsugu et al., 2013). The virtual environment stimulation method is mature and had been proved scientific and feasible by many researchers (Gall et al., 2021; Mollazadeh and Zhu, 2021; Capatina et al., 2024). Although the process and mechanism of virtual natural environment stimulation and real experience had not been thoroughly compared and demonstrated, they had certain similarities in terms of their effects on health (Li et al., 2023; Wen et al., 2024). A simulated nature experience has been shown to attract young people to digital media over outdoor recreation (Pergams and Zaradic, 2006) and to facilitate the rotection of seniors from environmental hazards and barriers (Satariano et al., 2012; Wen et al., 2018). Crucially, human-nature connections can be enhanced by exposure to simulated natural environments, and people may be more inclined to visit their actual counterparts (Litleskare et al., 2020; Chan et al., 2023). At the same time, virtual nature experience had been proved to increase real pro-environmental behaviors (Ibanez and Roussel, 2022; Spangenberger et al., 2024), and thus obtain health benefits through the real natural environment.

Currently, there is a relatively limited amount of research on the restorative aspects of virtual UGSs involving multisensory stimulation. The human senses of visual, audition, olfaction, tactile, and gustation are interconnected and mutually constraining (Schreuder et al., 2016). Only a few studies have focused on various sensory combinations that promote restoration and the quality of environmental perception (Hedblom et al., 2019; Zhang et al., 2019). Visual and auditory are the sensory elements that scholars initially considered. Research indicates that the accessibility of visual information influences the auditory perception of both artificial and natural sounds, while the accessibility of auditory information affects the perception of various visual elements (Jeon and Jo, 2020). Scholars have analyzed the characteristics of the interactive effects between vision and hearing using methods such as 3D virtual technology, suggesting that visual and auditory information are consistent. The most valuable sensory combination is vision and hearing, with vision taking the lead (Lindquist, 2014; Lindquist and Lange, 2014). The research have explored the physiological and psychological responses of individuals to combined visual and auditory stimuli, such as bird songs, confirming that people experience actual restoration from bird songs and associated landscapes (Ratcliffe et al., 2013). Through a combined audio-visual study, Zhao et al. found that the presence of birdsong enhances the psychological recovery potential of green spaces (Zhao et al., 2018). Deng et al. showed that integrating natural sounds such as birdsong into visual scenes of UGSs can enhance psychological recovery (Deng et al., 2020). In terms of research on the impact of environmental perception quality evaluation, Viollon et al. found that among eight types of urban soundscapes, the birdsong soundscape had the greatest impact on the visual degree of urbanization (Viollon et al., 2002).

On this basis, several scholars have gradually incorporated olfaction into multisensory empirical research. Ulrich suggested that many smells and sounds in the natural environment affect people’s feelings and emotions (Ulrich, 1983). Through indoor simulation experiments, several scholars have verified that the addition of olfactory stimulation increases the restorative potential of the original audio-visual environment (Sona et al., 2019). The differentiated outcomes of additional sensory combinations have led scholars to think about more complex interactions between multiple sensory stimuli. Several studies have shown a masking effect between auditory and olfactory stimuli. When one stimulus is strong, the perceived intensity of the other stimulus is weak. For example, an increase in hearing reduces the perception of smell, but an increase in smell increases the perception of hearing (Wang et al., 2022). Ba and Kang suggested that the type of odor, concentration of the odor, and different bird song volumes jointly affect overall comfort and consistency (Ba and Kang, 2019). In addition, Song et al. conducted a physiological-psychological recovery study based on a forest scene, and the results showed that, compared with other stimuli, the visual-olfactory stimuli combination played a more effective role in the control group (Song et al., 2021). Although scholars have focused on three sensory factors (bird songs, visual natural scenes, and plant smells) (Hedblom et al., 2019; Sona et al., 2019), the restorative effects of bird songs and other sensory combinations have not been compared, limiting their use in terms of reference. In the study by Song et al., the ln (LF/HF) values (representing the ratio of low-frequency to high-frequency power, indicative of heart rate variability) suggested that a single visual stimulus exhibits a greater recovery trend than a visual-olfactory stimuli combination (Song et al., 2019, 2021). In addition, scholars have conducted internet surveys and simulated 3D experiments using three parameters—functional, physiological, and subjective evaluations—to investigate the relationships between auditory and olfaction in the landscapes of pocket parks and their impacts on human health (Bitterman and Simonov, 2017). They argued that the use of a multisensory experience design in urban pocket parks contributes to improving quality of life, promoting relaxation, and alleviating work-related stress for urban residents (Bitterman and Simonov, 2017). Touch and taste are rarely involved in simulating stimuli, but studies have shown the tactile sensations produced through contact with plants in green spaces, along with the gustatory characteristics of plant branches, leaves, and fruits, have effects on the mental health of consumers, thereby influencing people’s experiences in environments (Ziegler, 2015).

In summary, research on the relationship between multisensory experiences and the restorative effects of UGSs has focused mainly on vision and hearing, with less research on the other senses. Moreover, when sensory stimulation is complex, it is not just a simple superposition of beneficial relationships. Physical access to nature is sometimes restricted by various factors, resulting in urbanities being deprived of the opportunity to enjoy nature (Bratman et al., 2019). In addition, the elderly, supervised personnel, patients and other people with impaired mobility also need the stimulation of virtual natural environment to restore physical and mental health (Nadkarni et al., 2021; Li et al., 2023; Wen et al., 2024). At the same time, the rise of the meta-universe also provides a higher possibility of obtaining health effects through virtual natural environment experiences (Capatina et al., 2024). It is necessary to explore the interaction and restoration effect of different sensory stimuli in virtual urban green space environment.

Therefore, with vision-audition as the basic sense, this study explored the differences in the impact of different virtual sensory stimulation in UGSs on the recovery of physiological and psychological health based on eight sensory combinations: the visual–auditory (VA), visual–auditory-olfactory (VAO), visual–auditory-tactile (VAT), visual–auditory-gustatory (VAG), visual–auditory-olfactory-tactile (VAOT), visual–auditory-olfactory-gustatory (VAOG), visual–auditory-tactile-gustatory (VATG), and visual–auditory-tactile-olfactory-gustatory (VATOG) stimuli combinations.

## 2 Materials and methods

### 2.1 Participants

Posters were placed around campus to recruit participants with the following characteristics: (1) self-reported normal vision and hearing, (2) no physical or mental illness, and (3) not taking any medications. Participants were asked to avoid smoking, drinking, and strenuous physical activity throughout the study period. Kotabe et al. proposed that the ratings of 20 participants are sufficient to obtain reliable image evaluation (Ziegler, 2015). To improve the reliability and validity of the experimental results, 240 volunteers (50% male; 50% female, average age 21.3 ± 2.8 years) were ultimately recruited for the study. There were a total of 8 experimental groups in this study (the VA group [T1]; VAO group [T2]; VAT group [T3]; VAG group [T4]; VAOT group [T5]; VAOG group [T6]; VATG group [T7]; and VATOG group [T8]). To control for the interference of gender on the results, volunteers of both genders were randomly assigned experimental serial numbers ranging from 1 to 120 and were assigned to each experimental group in order, ensuring that each experimental group included 30 people (15 males and 15 females). The study was performed with the approval of the local Ethics Committee of the College of Landscape Architecture, Sichuan Agricultural University, China.

### 2.2 Sensory stimulation materials

**Figure 1:**
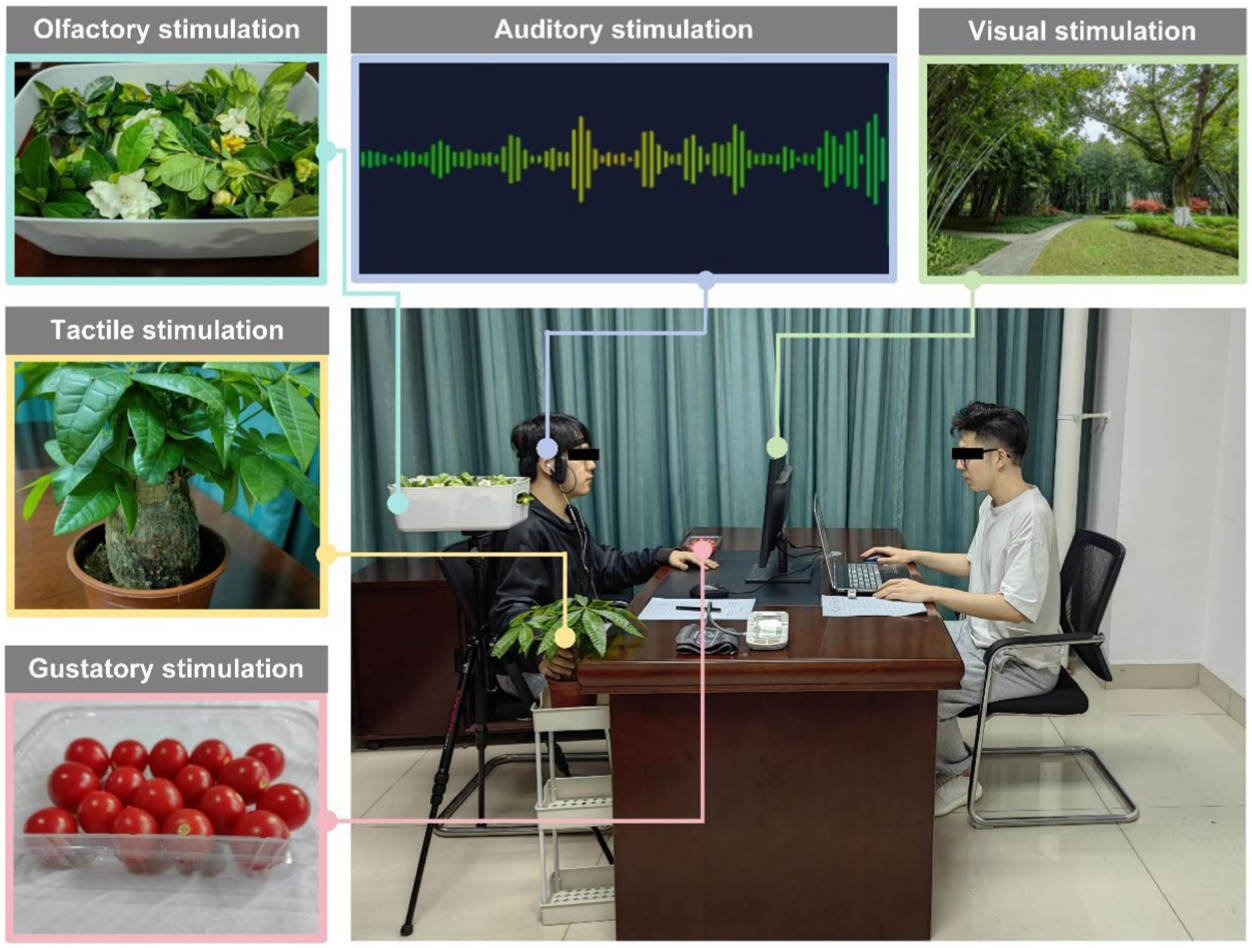
Experiment with five sensory stimuli (visual, auditory, olfactory, tactile, and gustatory).

The experiment involved a total of five sensory stimuli, as depicted in the Figure 1 below. A detailed description of each stimulus is provided in the following text:

#### 2.2.1 Visual stimulation materials

As visual materials of real landscapes, images are widely used in research because of the ability to effectively control for confounding variables (Cañas et al., 2009). This research method ensures that participants’ subjective judgements are not affected by weather, temperature, background sounds, or human activities in the real environment (Wang et al., 2017; Deng et al., 2020). This study utilized real-world photographs of UGSs in Chengdu, Sichuan Province, China, taken with a SONY DSLR-A350 camera as the visual stimuli. The photographs were captured from the normal perspective of an individual to simulate the human visual experience. To ensure the representativeness of the samples, a total of 28 sites were selected to be photographed. Initially, 10 images were taken at each site, resulting in 280 images for preliminary screening. Five experts evaluated these images, and ultimately, 25 images were selected as the formal experimental materials. The complete visual material is detailed in the Supplementary material. During the formal experiment, the visual stimuli were automatically switched every 12 s, for a total duration of 5 min.

#### 2.2.2 Auditory stimulation materials

The auditory stimuli were natural sounds collected simultaneously during the abovementioned visual material collection process. Birdsong, running water, and the rustling of leaves in the wind were the three main sound stimuli collected. Recording equipment was used to record three types of sounds in the environment. The recording equipment was 1.5 m from the ground. The duration of each audio recording was no less than 1 min. The recorded audio was imported into Adobe Audition 2022 software for extraction, separation, noise reduction, merging, loudness matching, and the adjustment of sound pressure levels. The resulting audio files (MP3) for the three types of sounds were subsequently exported. Afterward, Adobe Audition 2022 software was used to integrate the audio material, synchronizing it with the visual material. For example, the sound of flowing water could only be synchronized with images containing water scenes, and the intensity was adjusted based on the scene. To ensure that participants could clearly hear the sounds in the laboratory without feeling annoyed, the sound pressure level was set between 40 and 60 dBA with a step size of 5 dBA. A subjective loudness preexperiment was conducted with 10 volunteers who rated the perceived loudness on a scale from 1 to 5 (ranging from very quiet to very noisy). The results indicated that a sound pressure level of 55 dB was clear but not ear-piercing for all the sound stimuli. The final experimental audio material was adjusted within this loudness and sound pressure level range, with a duration totaling 5 min.

#### 2.2.3 Olfactory stimulation materials

The olfactory stimulation material used was *Gardenia jasminoides*, a shrub commonly found in the environment. *Gardenia jasminoides* belongs to the *Rubiaceae* family and is a deciduous shrub with fragrant flowers, typically borne singly at the top of the branches. The collected branches were placed in an opaque box for 5 min before the experiment to allow fragrance volatilization (Qi et al., 2022). Following the pretest assessment, a tripod was used to position the branches parallel to and approximately 30 cm from the side of the participant’s face, as shown in Figure 1. This placement allowed participants to smell the fragrance while avoiding any peripheral visual contact with the plant. This method facilitated more efficient release of the plant’s fragrance and eliminated psychological cues resulting from visual stimuli.

#### 2.2.4 Tactile stimulation materials

The tactile stimulation material used for this experiment was a potted *Pachira glabra Pasq.* plant. These plants have heights ranging from 8 to 15 cm, with an upright stem that is slightly rough. The plants exhibit palmate compound leaves with 5–7 leaflets, shaped like elongated ovals or inverted eggs, with each leaflet measuring 12–15 cm in length and approximately 6 cm in width (as shown in Figure 1). The plant’s stem feels similar to that of the trunks of common trees found in UGSs; the plant is not prickly and can easily be touched. These characteristics align with the experimental requirements for activating the sense of touch. In the experimental groups involving tactile stimulation, the participants were reminded before the experiment that they could touch the plants spontaneously during the experiment.

#### 2.2.5 Gustatory stimulation materials

The gustatory material used in this study was the cherry tomato, which is a natural fruit. It tastes sour and sweet and can effectively activate the sense of taste. Prior to the experiment, a preliminary screening of taste stimuli was conducted to ensure uniformity in size, color, and appearance. Random tastings were performed to guarantee a consistent taste experience. During the formal experiment, the cleaned fruits were placed in transparent, simple containers (as shown in Figure 1). In the experimental group involving gustatory stimuli, the participants were informed before the experiment that they were free to spontaneously consume the fruits during the experiment.

### 2.3 Measurements

#### 2.3.1 Physiological indicators

Blood pressure (systolic blood pressure [SBP; mmHg], diastolic blood pressure [DBP; mmHg], pulse [P; bpm]) was measured on the left arm before and after the experiment using a sphygmomanometer (Omron, HEM-6322T, Tokyo, Japan). To minimize errors, two measurements were taken both before and after the experiment, and the average was calculated as the final result. Blood pressure and pulse rate are considered indicators of the body’s state of arousal or relaxation. During periods of tension, both the SBP and DBP tend to increase, whereas they decrease during relaxation. An increase in pulse occurs during exercise or during emotional fluctuations.

#### 2.3.2 Psychological indicators

The Profile of Mood States (POMS) was utilized as the psychological tool in this study. The POMS consists of 40 adjectives rated on a scale from 0 to 4 (“0” indicates “not at all,” and “4” indicates “extremely”). These adjectives can be consolidated into seven emotional dimensions: tension-anxiety (T-A), depression (D), anger- hostility (A-H), vigor (V), fatigue (F), confusion (C), and self-esteem (S). Three psychological indicators—positive mood (PM), negative mood (NM), and total mood disturbance (TMD)—can be calculated from the 40 adjective scores (Uysal et al., 2016). The complete POMS can be found in the Supplementary material.

### 2.4 Study sites

The experimental location was selected in the Landscape Laboratory of Sichuan Agricultural University. During the experiment, the room temperature was controlled at 25°C through the air conditioning system. The experimental location was divided into a pretest laboratory and a formal laboratory. The two laboratories were adjacent and had the same spatial layout. The subjects entered the formal laboratory after completing the pretest. The difference between the two laboratories was that in the formal laboratory, the corresponding stimulation materials were placed according to the actual stimulation, while in the pretest laboratory, only pretest indicators were measured. This method ensured that all participants completed the experiments in a uniform environment and eliminated interference from factors such as residual odors in the laboratory. The participants were informed of the entire process and purpose of the experiment in advance and signed an informed consent form before the experiment.

### 2.5 Procedure

**Figure 2:**
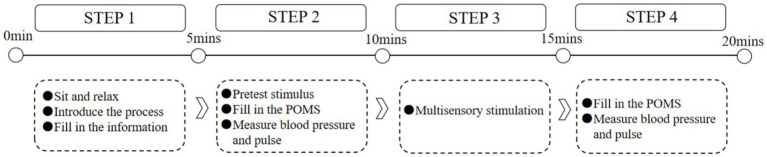
Flowchart of the experiment.

The experiment took place in April 2023 and spanned more than 8 days. During the experiment, the doors and windows were closed, the blackout curtains were drawn, and indoor lighting was uniformly turned on to control the illumination environment. The room temperature was regulated through an air conditioning system. The experiment was divided into four main sections, and the whole experiment lasted approximately 20 min, as illustrated in Figure 2.

**Figure 3:**
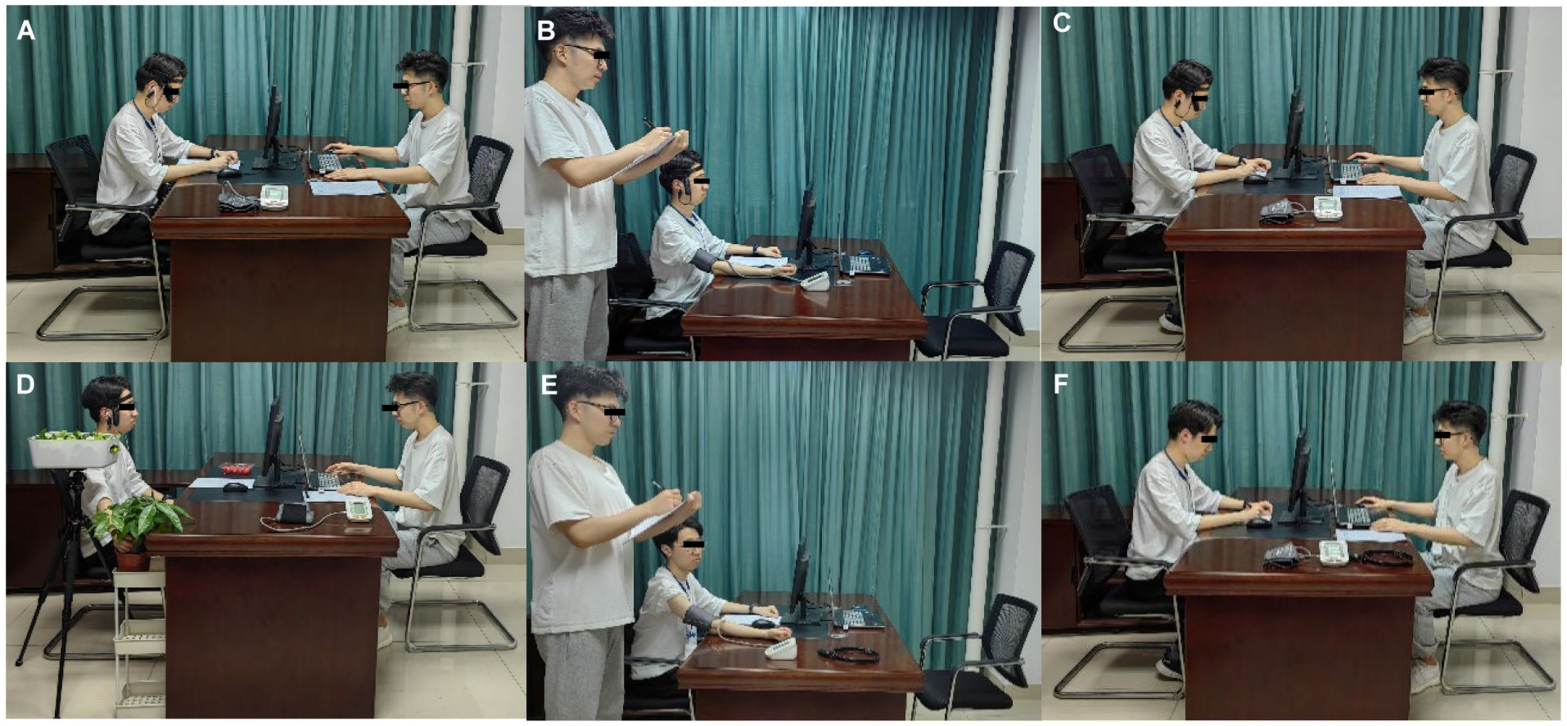
Schematic diagram of the experimental procedure [**(A)** pretest stimulus (noise + calculation); **(B)** Blood pressure and pulse measurements before the experiment; **(C)** Measurement of psychological indicators before the experiment; **(D)** sensory stimulation; **(E)** Blood pressure and pulse measurements after the experiment; **(F)** Measurement of psychological indicators after the experiment].

The first part involved collecting basic information from participants and informing them in advance about the purpose and details of the study to help them understand the experiment and minimize errors during data collection. Participants were then instructed to sit quietly and relax for 3 min to reduce the impact of other states before the experiment. The second part was the pretest, where participants entered the pretest laboratory, wore headphones, listened to noisy audio for 5 min, and simultaneously performed mathematical calculations. After the pretest, participants completed the POMS, and their blood pressure and pulse were measured. The pretest results for all eight experimental groups were identical, and the tests were conducted in the same environment. The third part involved sensory stimulation. The corresponding stimulation materials for visual, auditory, olfactory, tactile, and gustatory stimuli were arranged in the formal laboratory in advance according to the treatment of the different experimental groups. Research has shown that contact with a natural environment for 3–5 min can provide a sufficient restorative experience (Deng et al., 2020). Therefore, after entering the formal laboratory, the subjects were exposed to sensory stimuli collected from UGSs for 5 min and underwent the related experiences described in Section 2.2. Notably, only one type of treatment group experiment was conducted each day. After each experimental day, thorough cleaning and ventilation were carried out to ensure that no residual odors or other interfering factors remained in the environment. This practice was followed before starting the experiments for the other treatment groups the next day, eliminating interference from lingering odors or other materials. The fourth part involved posttest measurements. After exposure to sensory stimuli, participants underwent another assessment of their psychological state using the POMS, along with the measurement and recording of their blood pressure and pulse. The complete experimental process is illustrated in Figure 3.

### 2.6 Statistical analyses

Paired *T*-test was used to analyze the differences of physiological and psychological indicators before and after the experiment, and the difference data before and after each indicator met the normal distribution as a whole, meeting the prerequisites of paired *T*-test. One-way analysis of variance (ANOVA) was used to compare the difference between groups of physiological and psychological indicators before and after the experiment. The data of each group met the homogeneity of variance and there were significant differences between the groups. The *post hoc* multiple test was performed by Bonferroni test. All the statistical analyses were conducted using SPSS 24.0 (SPSS, Chicago, IL, USA). In this study, a *p-*value <0.05 was considered to indicate statistical significance.

## 3 Results

### 3.1 Physiological recovery

**Figure 4:**
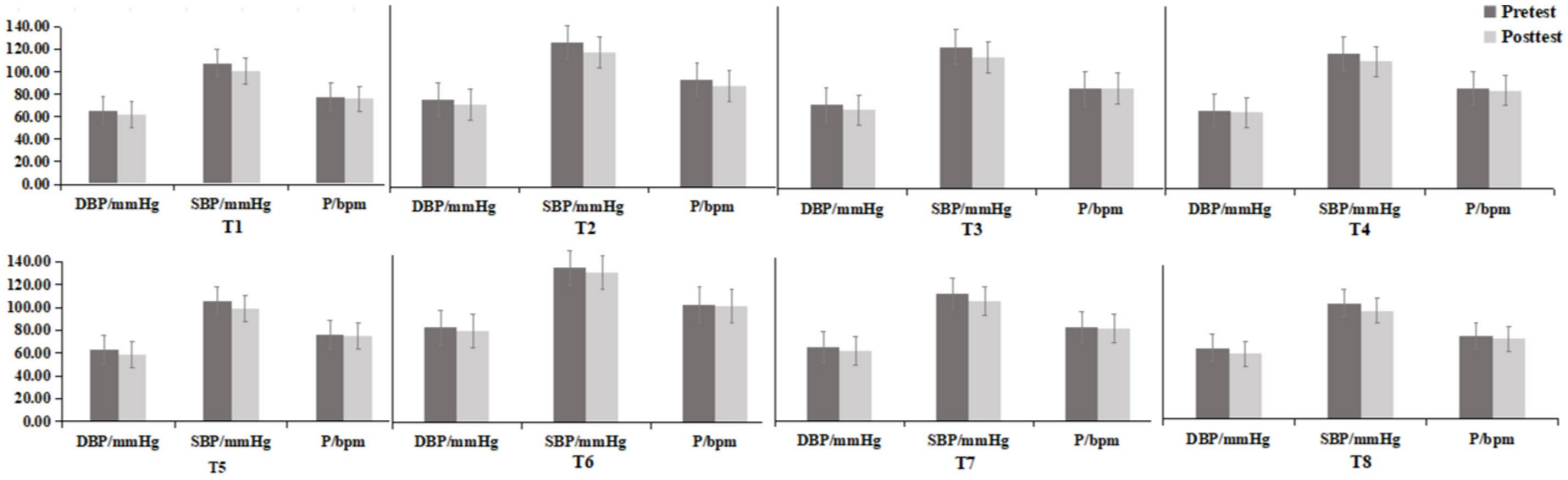
Blood pressure and pulse index results for each group (mean ± standard deviation).

The physiological indicators before and after the experiment for the eight experimental groups are presented in Figure 4. After the environmental intervention, the blood pressure values for each experimental group decreased, while the pause values slightly decreased or remained relatively constant compared to the pretest values.

**Figure 5:**
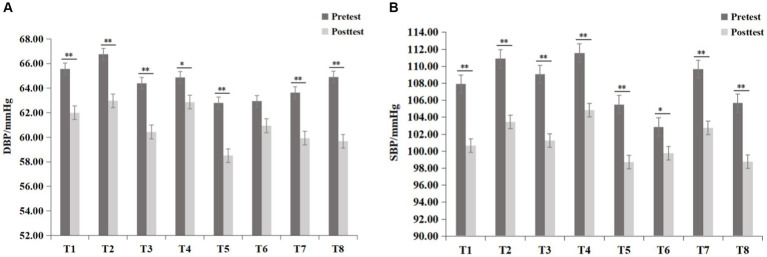
Difference of blood pressure among groups (Mean ± standard deviation; *p < 0.05, **p < 0.01; **(A)** Diastolic blood pressure [DBP], **(B)** Systolic blood pressure [SBP]).

**Figure 6:**
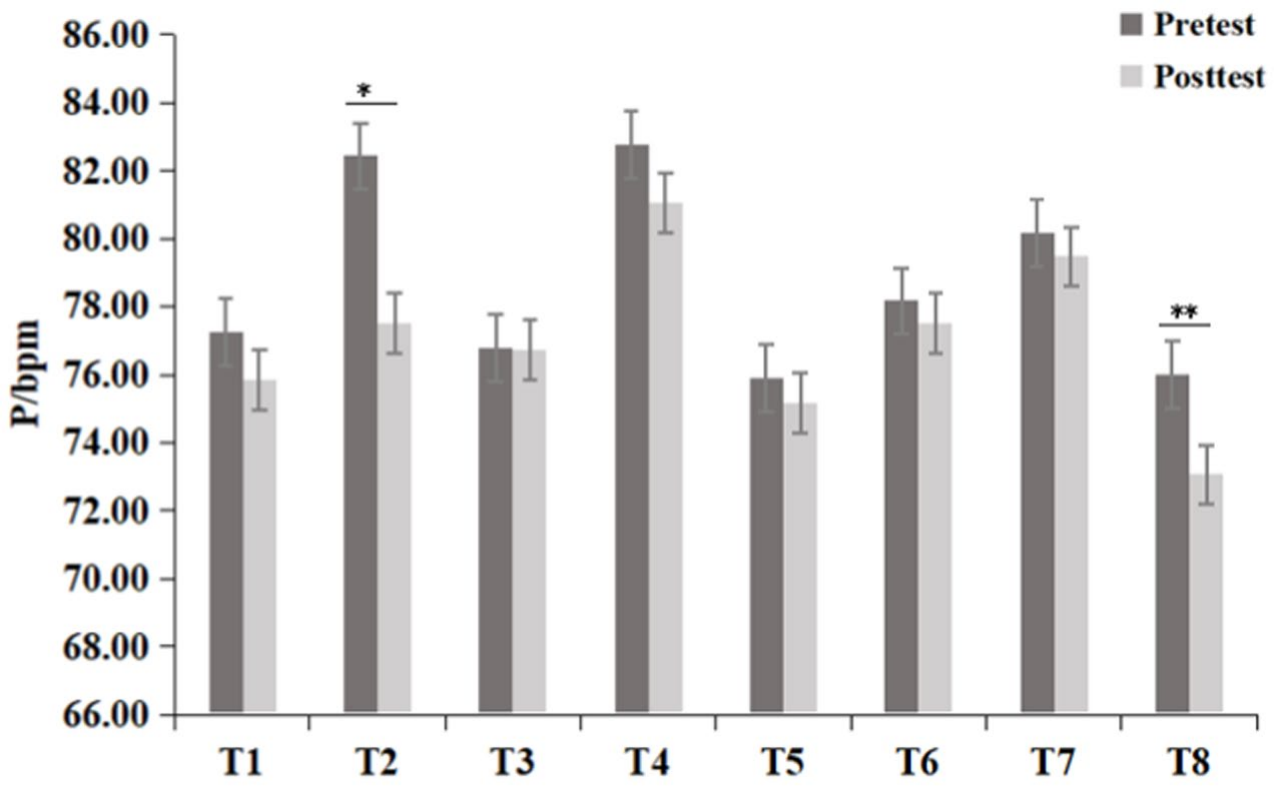
Differences in pulse (P) among the groups (means ± standard deviations; *p < 0.05, **p < 0.01).

To determine whether the differences between groups were objective, further statistical tests were conducted on the data of each group. The differences between the groups conformed to the homogeneity of variances and were normally distributed, meeting the prerequisites of the paired t test. As shown in Figure 5, the SBP of all eight experimental groups significantly decreased after the experiment (*p* < 0.05). There was no significant difference in the DBP of the T3 group before and after the experiment, but the other seven experimental groups exhibited a significant decrease in DBP (*p* < 0.05). The pulse decreased in all groups, but a significant decrease was observed only in the T3 group (*t* = −2.18, *p* = 0.037) and T8 group (*t* = −3.15, *p* = 0.004), as shown in Figure 6.

Specifically, as shown in Table 1, the greatest decrease in DBP was observed in the T8 group (−5.23 ± 0.78 mmHg) and the second greatest decrease was observed in the T5 group (−4.23 ± 0.85 mmHg). DBP decreased the least in the T4 group (−2.00 ± 0.97 mmHg), and the second-lowest decrease was observed in the T1 group (−3.57 ± 1.02 mmHg). The decreases in DBP were similar in the remaining groups: the T2 group (−3.80 ± 0.90 mmHg), T3 group (−3.97 ± 0.93 mmHg), and T7 group (−3.70 ± 0.72 mmHg). The decrease in SBP was the largest in the T3 group (−7.80 ± 0.97 mmHg) and the second largest in the T2 group (−7.47 ± 1.30 mmHg), and the decrease in the T1 group was similar (−7.27 ± 1.05 mmHg). The decrease in SBP was the lowest in the T6 group (−3.10 ± 1.38 mmHg), and the decreases in the other groups were similar, ranging from 6.73 to 6.90 mmHg. The T2 group exhibited a greater decrease in the pause rate (−4.93 ± 2.26 bpm) than the T8 group (−2.93 ± 0.93 bpm).

**Table 1:** Paired *T*-test results of blood pressure and pulse samples.

Test group	Indicators	Difference (post-pre)	SD	SE	95%CI		*t*	*p*
					Lower	Upper		
T1	DBP/mmHg	−3.57	5.58	1.02	−5.65	−1.49	−3.50	0.002**
	SBP/mmHg	−7.27	5.77	1.05	−9.42	−5.11	−6.90	0.000**
	P/bpm	−1.40	6.07	1.11	−3.67	0.87	−1.26	0.216
T2	DBP/mmHg	−3.80	4.91	0.90	−5.63	−1.97	−4.24	0.000**
	SBP/mmHg	−7.47	7.12	1.30	−10.13	−4.81	−5.75	0.000**
	P/bpm	−4.93	12.38	2.26	−9.56	−0.31	−2.18	0.037*
T3	DBP/mmHg	−3.97	5.08	0.93	−5.86	−2.07	−4.28	0.000**
	SBP/mmHg	−7.80	5.31	0.97	−9.78	−5.82	−8.05	0.000**
	P/bpm	−0.03	7.47	1.36	−2.82	2.76	−0.02	0.981
T4	DBP/mmHg	−2.00	5.30	0.97	−3.98	−0.02	−2.07	0.048*
	SBP/mmHg	−6.73	5.17	0.94	−8.66	−4.80	−7.14	0.000**
	P/bpm	−1.70	5.51	1.01	−3.76	0.36	−1.69	0.102
T5	DBP/mmHg	−4.30	4.64	0.85	−6.03	−2.57	−5.08	0.000**
	SBP/mmHg	−6.77	5.04	0.92	−8.65	−4.88	−7.35	0.000**
	P/bpm	−0.73	6.05	1.11	−2.99	1.53	−0.66	0.512
T6	DBP/mmHg	−2.00	5.98	1.09	−4.23	0.23	−1.83	0.077
	SBP/mmHg	−3.10	7.54	1.38	−5.92	−0.29	−2.25	0.032*
	P/bpm	−0.67	5.31	0.97	−2.65	1.32	−0.69	0.497
T7	DBP/mmHg	−3.70	3.91	0.72	−5.16	−2.24	−5.18	0.000**
	SBP/mmHg	−6.90	7.98	1.46	−9.88	−3.92	−4.74	0.000**
	P/bpm	−0.70	5.57	1.02	−2.78	1.38	−0.69	0.497
T8	DBP/mmHg	−5.23	4.26	0.78	−6.83	−3.64	−6.72	0.000**
	SBP/mmHg	−6.90	6.26	1.14	−9.24	−4.56	−6.04	0.000**
	P/bpm	−2.93	5.10	0.93	−4.84	−1.03	−3.15	0.004**

*N = 240, *n* = 30. DBP, Diastolic blood pressure; SBP, Systolic blood pressure; P, Pulse. *p < 0.05, **p < 0.01.

**Figure 7:**
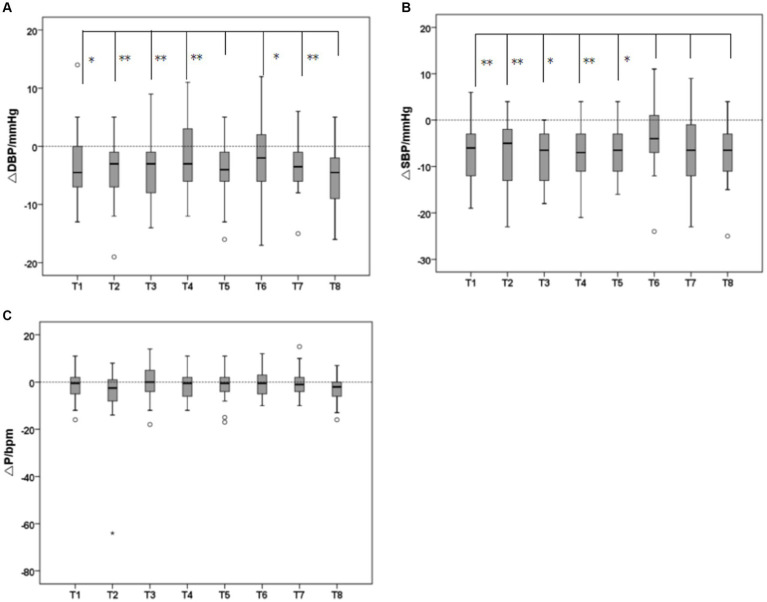
Results of variance analysis of recovery effects of physiological indicators between groups [**(A)** Diastolic blood pressure (DBP), **(B)** Systolic blood pressure (SBP), **(C)** Pulse (P); *p < 0.05, **p < 0.01].

As shown in Figure 7, the recovery difference results of physiological indicators between groups showed that there were significant differences between VAOT (T5) and VAOTG (T8) recovery effects and the other groups (*p* < 0.05), and the reduction rate was significantly higher than that of the other groups, indicating that olfactory-tactile interaction can significantly increase the recovery of DBP. The recovery effect of VATG (T7) and VAOTG (T8) was significantly different from that of other groups (*p* < 0.05), and the decline rate was significantly higher than that of other groups, indicating that tactile -gustatory interaction could significantly increase the recovery of SBP, while the difference was not significant among other groups. The recovery effect of VAOG (T6) was significantly different from that of the other groups (*p* < 0.05), and the decrease rate was significantly lower than that of the other groups, indicating that olfactory-tactile interaction could reduce the recovery effect of systolic blood pressure (still had a recovery effect), and the difference between the other groups was not significant. There were no significant differences in pulse recovery between groups. The result of the variance model of physiological index recovery between groups was shown in Table 2. The result of multiple comparisons of Bonferroni for more specific inter-group differences was shown in Table 3.

**Table 2:** Results of ANOVA of physiological indicators recovery between groups.

Indicators		Sum of squares	df	Mean Square	*F*	*p*
DBP	Between groups	1503.600	7	214.800	11.412	0.000*
	Within groups	4366.733	232	18.822		
	Total	5870.333	239			
SBP	Between groups	1706.729	7	243.818	12.450	0.000*
	Within groups	4543.567	232	19.584		
	Total	6250.296	239			
P	Between groups	534.429	7	76.347	1.534	0.157
	Within groups	11549.033	232	49.780		
	Total	12083.463	239			

*The mean difference is significant at the 0.05 level. *N* = 240, *n* = 30. DBP, diastolic blood pressure; SBP, systolic blood pressure; P, pulse.

**Table 3:** The results of the Bonfreni Multiple Comparisons on the difference between the recovery groups of physiological indicators.

Indicators	(I) Test group (post-pre)	(J) Test group (post-pre)	Mean difference (I-J)	SE	*p*	95%CI	
						Lower	Upper
DBP/mmHg	T5	T1	−1.133*	1.120	0.012	−7.54	−0.46
		T2	−1.100*	1.120	0.004	−7.87	−0.79
		T3	−1.267*	1.120	0.008	−7.67	−0.59
		T4	−1.700*	1.120	0.000	−9.64	−2.56
		T6	−1.700*	1.120	0.000	−9.67	−2.59
		T7	−1.600*	1.120	0.001	−8.34	−1.26
		T8	1.533	1.120	1.000	−2.44	4.64
	T8	T1	−1.667*	1.120	0.000	−8.64	−1.56
		T2	−1.433*	1.120	0.000	−8.97	−1.89
		T3	−1.267*	1.120	0.000	−8.77	−1.69
		T4	−3.233*	1.120	0.000	−10.74	−3.66
		T5	−1.533	1.120	1.000	−4.64	2.44
		T6	−3.233*	1.120	0.000	−10.77	−3.69
		T7	−1.933*	1.120	0.000	−9.44	−2.36
SBP/mmHg	T6	T1	4.167*	1.143	0.012	0.93	7.40
		T2	4.367*	1.143	0.008	1.13	7.60
		T3	4.700*	1.143	0.005	1.46	7.94
		T4	3.633*	1.143	0.028	0.40	6.87
		T5	3.667*	1.143	0.027	0.43	6.90
		T7	3.800*	1.143	0.022	0.56	7.04
		T8	3.800*	1.143	0.022	0.56	7.04
	T7	T1	−1.367*	1.143	0.002	−8.24	−1.02
		T2	−1.567*	1.143	0.000	−8.64	−1.42
		T3	−1.900*	1.143	0.000	−9.28	−2.06
		T4	−1.167*	1.143	0.001	−8.54	−1.32
		T5	−1.133*	1.143	0.001	−8.44	−1.22
		T6	−3.800*	1.143	0.000	−12.01	−4.79
		T8	0.000	1.143	1.000	−3.61	3.61
	T8	T1	−1.367*	1.143	0.002	−8.24	−1.02
		T2	−1.567*	1.143	0.000	−8.64	−1.42
		T3	−1.900*	1.143	0.000	−9.28	−2.06
		T4	−1.167*	1.143	0.001	−8.54	−1.32
		T5	−1.133*	1.143	0.001	−8.44	−1.22
		T6	−3.800*	1.143	0.000	−12.01	−4.79
		T7	0.000	1.143	1.000	−3.61	3.61

*The mean difference is significant at the 0.05 level. *N* = 240, *n* = 30. DBP, diastolic blood pressure; SBP, systolic blood pressure; P, pulse. There was no significant difference between the other groups.

### 3.2 Psychological recovery

**Figure 8:**
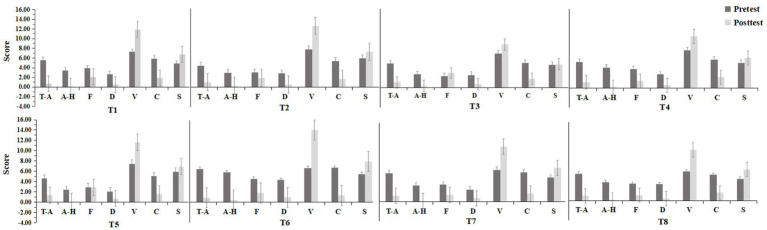
Results of the POMS for each group (T-A: tension-anxiety; A-H: anger- hostility; F: fatigue; D: depression; V: vigour; C: confusion; S: self-esteem).

The psychological indicator results of the pretest and posttest for the eight experimental groups are shown in Figure 8. After the experiment, the negative dimension psychological indicators (T-A, A-H, F, D, and C) decreased in all the experimental groups, while the positive dimension psychological indicators (V and S) increased.

**Figure 9:**
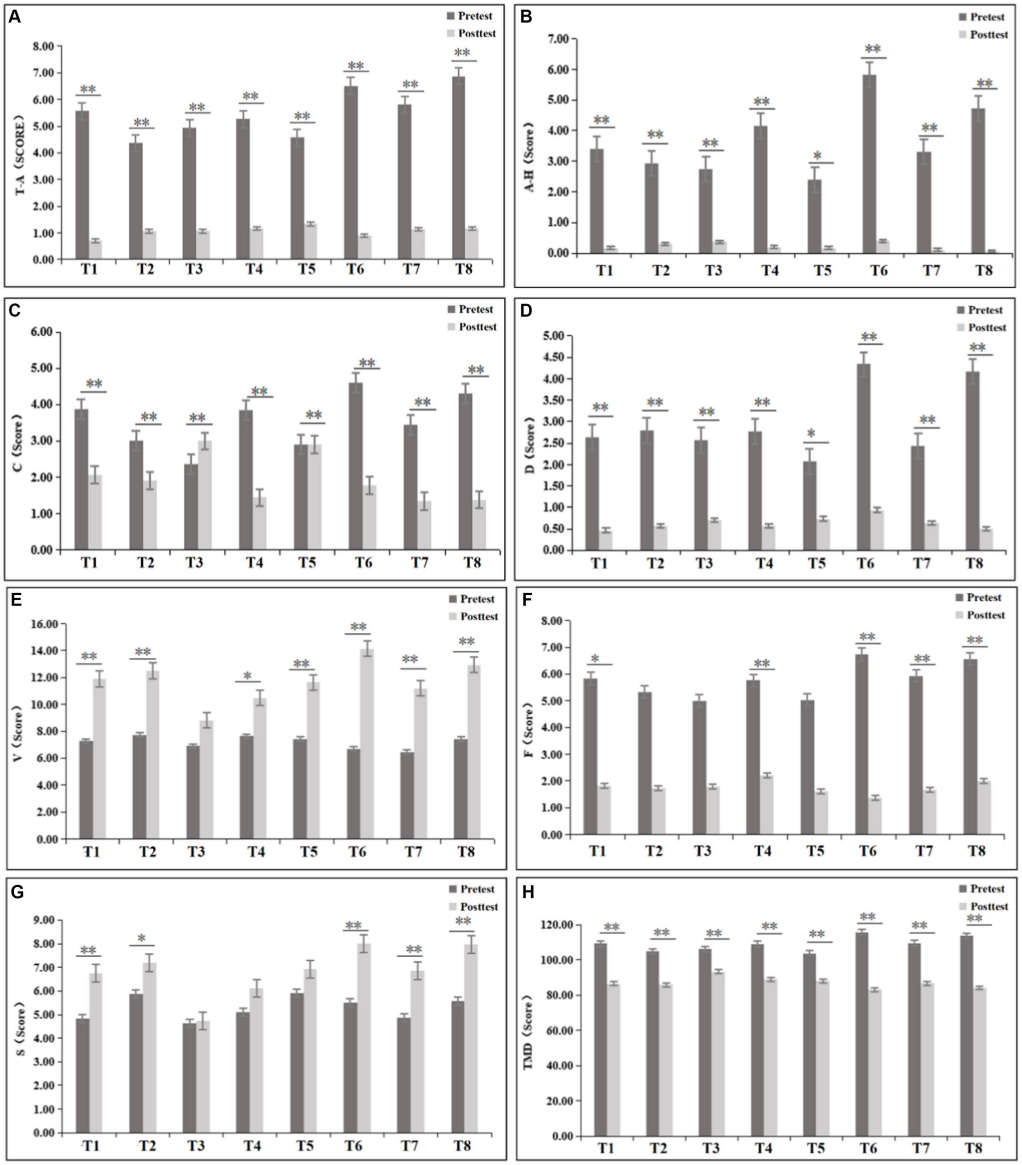
Results of different emotional dimensions in 8 experimental groups. [**(A)** tension-anxiety (T-A); **(B)** anger- hostility (A-H); **(C)** confusion (C); **(D)** depression (D); **(E)** vigor (V); **(F)** fatigue (F); **(G)** self-esteem (S); **(H)** total mood disturbance (TMD)]. *p<0.05, **p<0.01.

To ascertain whether the intergroup differences were significant, further statistical analysis was conducted on the data from each group. The data satisfied the homogeneity of variance assumption and had a normal distribution, meeting the prerequisites for paired *t-*tests. Paired *t-*tests were conducted on the pretest and posttest data for all variables within each group, and the results [as shown in Figure 9 indicated significant decreases (*p* < 0.05) in NM scores across the T-A, A-H, C, D dimensions, as well as in the TMD score, for all eight experimental groups after the experiment]. The F dimension score decreased in all eight experimental groups, with significant differences observed between groups, except for the T2, T3, and T5 groups (*p* < 0.05). Overall, these findings indicate that UGSs can effectively alleviate the public’s NM states. The V dimension score for PM increased in all eight groups after the experiment, with significant differences observed between groups, except for the T3 group (*p* < 0.05). Similarly, the S dimension score increased after the experiment in all eight groups, with significant differences observed between groups, except for the T3, T4, and T5 groups (*p* < 0.05). Overall, these findings suggest that UGSs can effectively enhance the PM states of the public.

Specifically, as shown in Table 4, within the T-A dimension, the values for all groups decreased, with the T8 group exhibiting the greatest reduction. The T6 and T7 groups had slightly greater reductions than did the T1 group, while the remaining four groups had lower reductions than did the T1 group, with the T5 group having the lowest reduction. In the A-H dimension, the values for all groups decreased, with the T6 group showing the greatest reduction. The T8 group ranked second in terms of reduction, followed by the T4 group. The reductions in the remaining three groups were similar to those in the T1 group. Concerning the C dimension, the values for the T1, T2, T4, T6, T7, and T8 groups all decreased. The T6 group had the greatest reduction, while the value for the T3 group increased, and the T5 group showed minimal changes. In the D dimension, the values for all the groups decreased, with the T6 and T8 groups exhibiting greater reductions. Within the V dimension, the values for all groups increased, with the most significant improvement observed in the T6 group. The T2 and T8 groups showed similar increases, which were slightly higher than that in the T1 group, while the increases in the remaining groups were lower than that in the T1 group. In the F dimension, the values for all the groups decreased, with greater decreases observed in the T6 and T8 groups. The T7 group ranked third in terms of reduction. Within the S dimension, the values for all groups increased, with notable improvements in the T6, T7, and T8 groups, all surpassing that for the T1 group. The improvements in the remaining groups were lower than that in the T1 group.

**Table 4:** Paired *T*-test results of POMS.

Test group	Indicators	Difference (post-pre)	SD	SE	95%CI		*t*	*p*
					Lower	Upper		
T1	T-A	−4.87	4.59	0.84	−6.58	−3.15	−5.81	0.000**
	A-H	−3.23	4.99	0.91	−5.10	−1.37	−3.55	0.001**
	F	−1.80	3.61	0.66	−3.15	−0.45	−2.74	0.011*
	D	−2.17	3.48	0.63	−3.46	−0.87	−3.42	0.002**
	V	4.63	6.97	1.27	2.03	7.24	3.64	0.001**
	C	−4.00	4.76	0.87	−5.78	−2.22	−4.61	0.000**
	S	1.93	3.48	0.64	0.63	3.23	3.04	0.005**
	TMD	−22.63	28.15	5.14	−33.15	−12.12	−4.40	0.000**
T2	T-A	−3.30	3.66	0.67	−4.67	−1.93	−4.94	0.000**
	A-H	−2.63	4.08	0.75	−4.16	−1.11	−3.53	0.001**
	F	−1.10	4.07	0.74	−2.62	0.42	−1.48	0.150
	D	−2.23	3.40	0.62	−3.50	−0.96	−3.60	0.001**
	V	4.77	6.46	1.18	2.36	7.18	4.04	0.000**
	C	−3.60	3.29	0.60	−4.83	−2.37	−6.00	0.000**
	S	1.33	3.03	0.55	0.20	2.47	2.41	0.023*
	TMD	−18.97	21.26	3.88	−26.91	−11.03	−4.89	0.000**
T3	T-A	−3.87	3.88	0.71	−5.31	−2.42	−5.47	0.000**
	A-H	−2.37	3.18	0.58	−3.55	−1.18	−4.08	0.000**
	F	0.63	3.75	0.68	−0.77	2.03	0.93	0.362
	D	−1.87	3.25	0.59	−3.08	−0.66	−3.15	0.004**
	V	1.90	6.34	1.16	−0.47	4.27	1.64	0.112
	C	−3.20	3.50	0.64	−4.51	−1.89	−5.01	0.000**
	S	0.10	2.64	0.48	−0.89	1.09	0.21	0.837
	TMD	−12.67	21.45	3.92	−20.68	−4.66	−3.24	0.003**
T4	T-A	−4.10	4.34	0.79	−5.72	−2.48	−5.17	0.000**
	A-H	−3.97	5.77	1.05	−6.12	−1.81	−3.77	0.001**
	F	−2.40	3.91	0.71	−3.86	−0.94	−3.36	0.002**
	D	−2.20	3.97	0.73	−3.68	−0.72	−3.04	0.005**
	V	2.83	6.77	1.24	0.30	5.36	2.29	0.029*
	C	−3.57	3.64	0.66	−4.92	−2.21	−5.37	0.000**
	S	1.00	4.75	0.87	−0.77	2.77	1.15	0.258
	TMD	−20.07	27.64	5.05	−30.39	−9.74	−3.98	0.000**
T5	T-A	−3.23	4.35	0.79	−4.86	−1.61	−4.08	0.000**
	A-H	−2.23	5.46	1.00	−4.27	−0.19	−2.24	0.033*
	F	0.00	5.09	0.93	−1.90	1.90	0.00	1.000
	D	−1.33	3.06	0.56	−2.47	−0.19	−2.39	0.024*
	V	4.20	6.76	1.23	1.68	6.72	3.40	0.002**
	C	−3.43	3.80	0.69	−4.85	−2.01	−4.95	0.000**
	S	1.03	3.73	0.68	−0.36	2.43	1.52	0.140
	TMD	−15.47	26.86	4.90	−25.50	−5.44	−3.15	0.004**
T6	T-A	−5.60	5.22	0.95	−7.55	−3.65	−5.87	0.000**
	A-H	−5.43	5.89	1.08	−7.63	−3.24	−5.05	0.000**
	F	−2.83	4.53	0.83	−4.52	−1.14	−3.43	0.002**
	D	−3.40	4.39	0.80	−5.04	−1.76	−4.24	0.000**
	V	7.43	5.56	1.02	5.36	9.51	7.32	0.000**
	C	−5.37	4.25	0.78	−6.95	−3.78	−6.92	0.000**
	S	2.50	3.55	0.65	1.17	3.83	3.86	0.001**
	TMD	−32.57	28.38	5.18	−43.16	−21.97	−6.29	0.000**
T7	T-A	−4.67	4.02	0.73	−6.17	−3.17	−6.36	0.000**
	A-H	−3.20	5.26	0.96	−5.17	−1.24	−3.33	0.002**
	F	−2.10	2.98	0.54	−3.21	−0.99	−3.87	0.001**
	D	−1.80	3.03	0.55	−2.93	−0.67	−3.25	0.003**
	V	4.73	6.03	1.10	2.48	6.99	4.30	0.000**
	C	−4.27	3.84	0.70	−5.70	−2.83	−6.08	0.000**
	S	2.00	3.24	0.59	0.79	3.21	3.38	0.002**
	TMD	−22.77	23.80	4.35	−31.65	−13.88	−5.24	0.000**
T8	T-A	−5.70	4.18	0.76	−7.26	−4.14	−7.47	0.000**
	A-H	−4.67	5.79	1.06	−6.83	−2.50	−4.41	0.000**
	F	−2.93	3.95	0.72	−4.41	−1.46	−4.07	0.000**
	D	−3.67	4.70	0.86	−5.42	−1.91	−4.27	0.000**
	V	5.53	6.13	1.12	3.25	7.82	4.95	0.000**
	C	−4.57	3.25	0.59	−5.78	−3.36	−7.71	0.000**
	S	2.40	3.76	0.69	1.00	3.80	3.50	0.002**
	TMD	−29.47	25.43	4.64	−38.96	−19.97	−6.35	0.000**

*N = 240, *n* = 30. T-A, tension-anxiety; A-H, anger- hostility; F, fatigue; D, depression; V, vigor; C, confusion; S, self-esteem; TMD, total mood disturbance. *p<0.05, **p<0.01.

**Figure 10:**
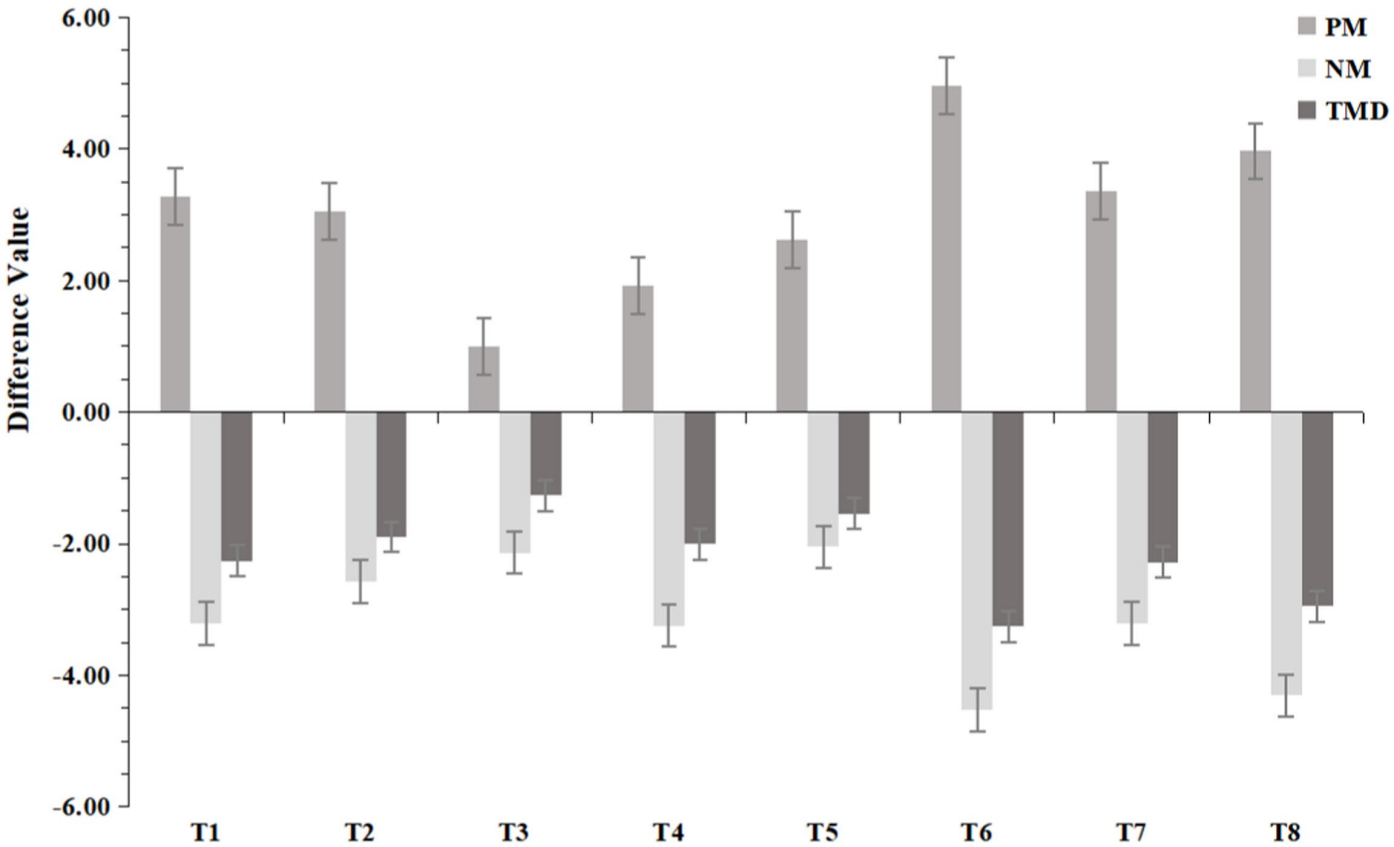
Results of the recovery of positive mood (PM), negative mood (NM) and total mood disturbance (TMD) in the 8 experimental groups.

The seven dimensions were integrated into two major dimensions: PM and NM. The overall TMD recovery results and them were compared and analyzed, as shown in Figure 10. The results indicated positive effects on enhancing PM and reducing NM in all eight experimental groups, and a positive recovery effect on the TMD score was observed (lower TMD values indicate better recovery). Specifically, within the PM dimension, the T6 group exhibited the largest improvement, with the T8 group ranking second. Similar improvements were observed in the T2 and T7 groups compared to the T1 group. The T3, T4, and T5 groups showed less improvement than the T1 group did, with the T3 group showing the least improvement. The overall patterns of decrease in the NM and TMD scores among the groups were similar to the patterns observed in the improvement in the PM score.

**Figure 11:**
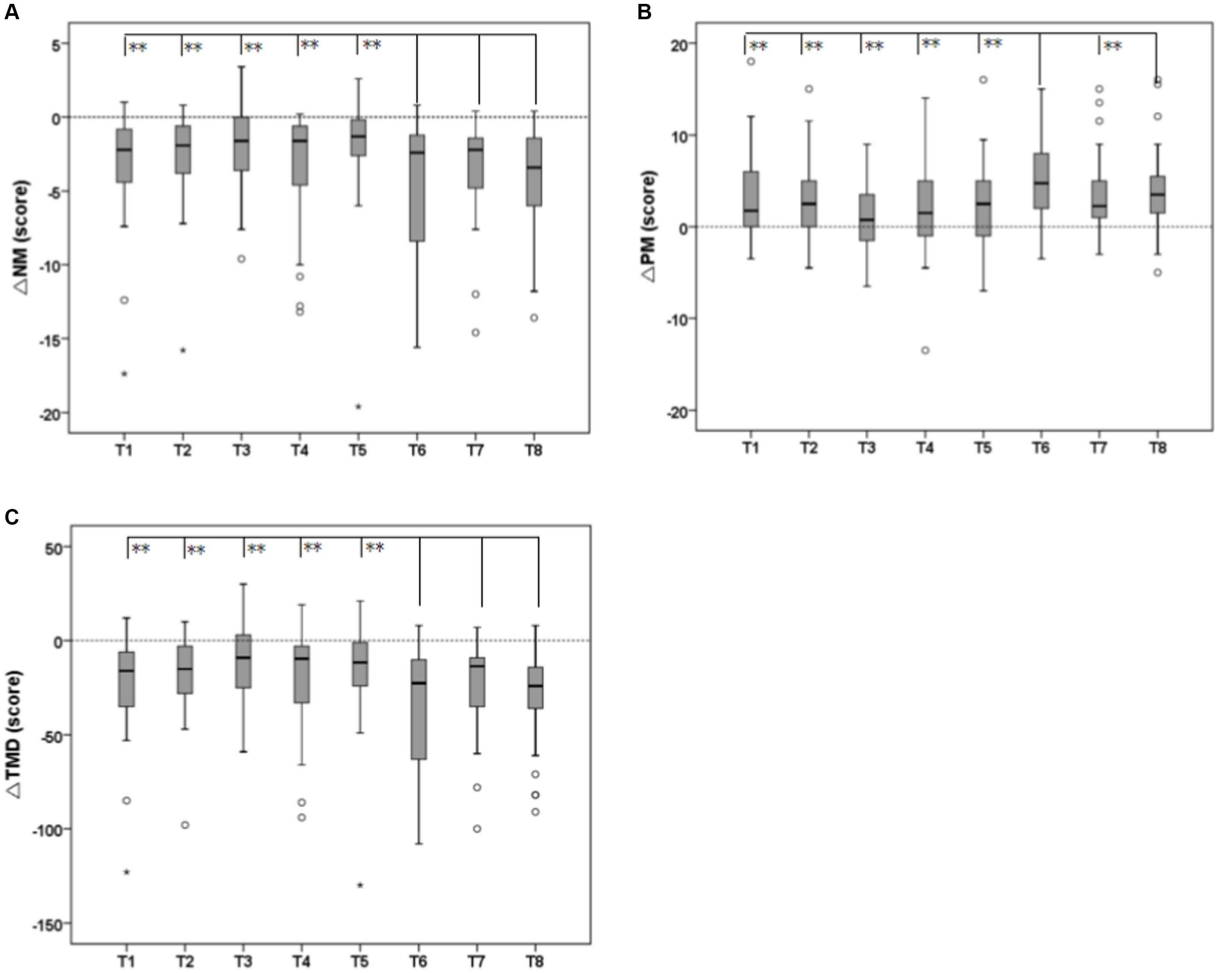
Results of variance analysis of recovery effects of psychological indicators between groups [**(A)** negative mood (NM); **(B)** positive mood (PM); **(C)** total mood disturbance (TMD); *p < 0.05, **p < 0.01].

As shown in Figure 11, the results show that the law of difference between NM and TMD groups is the same. The recovery effect of VAOG (T6), VAOTG (T7), and VAOTG (T8) is significantly different from that of other groups (*p* < 0.05), and the decline rate is significantly higher than that of other groups. These results indicate that olfactory-gustatory interaction can significantly increase the recovery effect of mental health, but there is no significant difference between other experimental groups. The difference between PM groups showed that the recovery effect of VAOG (T6) and VAOTG (T8) was significantly different from that of other groups (p < 0.05), and the increase rate was significantly higher than that of other groups, which also indicated that olfactory-gustatory interaction could significantly increase the recovery effect of mental health. There was no significant difference between other experimental groups. The result of the variance model of psychological index recovery between groups was shown in Table 5. The result of multiple comparisons of Bonferroni for more specific inter-group differences was shown in Table 6.

**Table 5:** Results of ANOVA of psychological indicators recovery between groups.

Indicators		Sum of squares	df	Mean square	*F*	*p*
NM	Between groups	2043.533	7	291.933	35.187	0.000*
	Within groups	1924.801	232	8.297		
	Total	3968.334	239			
PM	Between groups	1338.216	7	191.174	11.251	0.000*
	Within groups	3942.108	232	16.992		
	Total	5280.324	239			
TMD	Between groups	26313.462	7	3759.066	15.661	0.000*
	Within groups	55685.833	232	240.025		
	Total	81999.296	239			

*The mean difference is significant at the 0.05 level. *N* = 240, *n* = 30. PM, positive mood; NM, negative mood; TMD, total mood disturbance.

**Table 6:** The results of the Bonferroni Multiple Comparisons on the difference between the recovery groups of psychological indicators.

Indicators	(I) Test group (post-pre)	(J) Test group (post-pre)	Mean difference (I-J)	SE	*p*	95%CI	
						Lower	Upper
NM	T6	T1	−1.313*	0.744	0.000	−7.66	−2.96
		T2	−1.953*	0.744	0.000	−8.20	−3.50
		T3	−2.393*	0.744	0.000	−8.31	−3.61
		T4	−1.280*	0.744	0.000	−7.19	−2.49
		T5	−2.480*	0.744	0.000	−8.73	−4.03
		T7	−1.320	0.744	1.000	−2.57	2.13
		T8	0.220	0.744	1.000	−1.35	3.35
	T7	T1	−1.007*	0.744	0.000	−7.44	−2.74
		T2	−1.633*	0.744	0.000	−7.98	−3.28
		T3	−1.073*	0.744	0.000	−8.09	−3.39
		T4	−1.040*	0.744	0.000	−6.97	−2.27
		T5	−1.160*	0.744	0.000	−8.51	−3.81
		T6	1.320	0.744	1.000	−2.13	2.57
		T8	1.100	0.744	1.000	−1.13	3.57
	T8	T1	−1.093*	0.744	0.000	−8.66	−3.96
		T2	−1.733*	0.744	0.000	−9.20	−4.50
		T3	−2.173*	0.744	0.000	−9.30	−4.60
		T4	−1.060*	0.744	0.000	−8.19	−3.49
		T5	−2.260*	0.744	0.000	−9.72	−5.02
		T6	−0.220	0.744	1.000	−3.35	1.35
		T7	−1.100	0.744	1.000	−3.57	1.13
PM	T6	T1	1.683*	1.064	0.001	1.04	7.76
		T2	1.917*	1.064	0.003	0.87	7.60
		T3	3.967*	1.064	0.000	2.92	9.65
		T4	3.050*	1.064	0.000	2.37	9.10
		T5	2.350*	1.064	0.000	1.64	8.36
		T7	1.600*	1.064	0.002	0.99	7.71
		T8	1.000	1.064	1.000	−3.81	2.91
	T8	T1	0.683*	1.064	0.000	1.49	8.21
		T2	0.917*	1.064	0.000	1.32	8.05
		T3	2.967*	1.064	0.000	3.37	10.10
		T4	2.050*	1.064	0.000	2.82	9.55
		T5	1.350*	1.064	0.000	2.09	8.81
		T6	−1.000	1.064	1.000	−2.91	3.81
		T7	0.600*	1.064	0.000	1.44	8.16
TMD	T6	T1	−9.933*	4.000	0.000	−32.31	−7.02
		T2	−13.600*	4.000	0.000	−31.88	−6.59
		T3	−19.900*	4.000	0.000	−37.04	−11.76
		T4	−12.500*	4.000	0.000	−33.81	−8.52
		T5	−17.100*	4.000	0.000	−33.78	−8.49
		T7	−9.800	4.000	1.000	−17.61	7.68
		T8	3.100	4.000	1.000	−16.91	8.38
	T7	T1	−0.133*	4.000	0.008	−27.34	−2.06
		T2	−3.800*	4.000	0.012	−26.91	−1.62
		T3	−10.100*	4.000	0.000	−32.08	−6.79
		T4	−2.700*	4.000	0.002	−28.84	−3.56
		T5	−7.300*	4.000	0.002	−28.81	−3.52
		T6	9.800	4.000	1.000	−7.68	17.61
		T8	6.700	4.000	0.612	−3.41	21.88
	T8	T1	−6.833*	4.000	0.000	−36.58	−11.29
		T2	−10.500*	4.000	0.000	−36.14	−10.86
		T3	−16.800*	4.000	0.000	−41.31	−16.02
		T4	−9.400*	4.000	0.000	−38.08	−12.79
		T5	−14.000*	4.000	0.000	−38.04	−12.76
		T6	−3.100	4.000	1.000	−8.38	16.91
		T7	−6.700	4.000	0.612	−21.88	3.41

*The mean difference is significant at the 0.05 level. *N* = 240, *n* = 30. PM, positive mood; NM, negative mood; TMD, total mood disturbance. There was no significant difference between the other groups.

## 4 Discussion

### 4.1 Simulated multisensory stimulation and physiological recovery

The research findings revealed a significant decrease in SBP across all eight groups after the experiment. Except for the VAO group, the DBP significantly decreased in the remaining seven groups. Overall, these findings indicate that the sensory stimuli in all the groups had a certain restorative effect on physiological status, demonstrating that multisensory stimulation in UGSs can effectively alleviate public stress, which is consistent with prior research results (Zhao et al., 2018; Deng et al., 2020). Based on the foundation of VA stimuli, the individual addition of olfactory, tactile, or gustatory stimuli did not enhance the recovery of blood pressure. This finding aligns with previous studies (Ratcliffe et al., 2013; Zhao et al., 2018; Hedblom et al., 2019; Deng et al., 2020), as all eight sensory combinations had restorative effects on physiological health. The focus of discussion is on comparing the stronger restorative effects. Several studies have confirmed the beneficial role of olfaction in physiological recovery (Ulrich, 1983; Song et al., 2019, 2021). Olfactory stimulation is closely related to physiological recovery, such as pain alleviation and blood pressure regulation (Cha et al., 2010; Uysal et al., 2016). Overactivity in the sympathetic nervous system is one of the mechanisms of hypertension. Inhalation of odors through the nose can inhibit sympathetic nervous activity and reduce blood pressure. This may be one of the peripheral mechanisms underlying the blood pressure-lowering effects of olfactory stimulation (Haze et al., 2002; Tanida et al., 2006). However, intergroup comparisons between VA and VAO stimulation suggested that VAO stimulation is not necessarily superior to VA stimulation alone. This finding is consistent with the results of previous studies (Qi et al., 2022) and the viewpoint aligns with the idea that multiple sensory stimulation generates more complex outcomes than single or dual sensory stimulation (Hedblom et al., 2019). This study also provides effective guidance for future research in this field, suggesting a shift from primarily focusing on VAO stimuli to a more in-depth exploration of tactile and gustatory stimuli.

This study revealed that among the three groups treated with sensory stimulation via four modalities, the VAOT group (T5) and VATG group (T7) demonstrated better blood pressure recovery than did the VA group (T1). However, compared with the VA group (T1), in the VAOG group (T6), the blood pressure recovery effect was weaker, and the recovery effect in the VAOG group was also weaker than those in the VAO group (T2) and VAG group (T4). Taken together, these findings indicate that olfactory-gustatory stimuli interactions may diminish the recovery of blood pressure. For the physiological characteristic indicator of blood pressure, having more types of sensory stimuli does not necessarily result in better recovery. Furthermore, the recovery effects in the VAOT group (T5) were superior to those in the VAO group (T2) and VAT group (T3). Similarly, the VATG group (T7) exhibited better recovery than the VAT group (T3) and VAG group (T4). These findings collectively indicate that increasing sensory interaction involving olfaction-tactile and tactile-gustation stimuli can enhance physiological recovery on a VA basis. This further emphasizes that olfaction and gustation need to be coupled with tactile stimulation to produce optimal physiological recovery benefits.

Empirical research on the recovery effects of tactile stimulation on physiological indicators has been relatively scarce. However, some research has shown that C-tactile afferents may regulate oxytocin release during intimate tactile interactions, thereby reducing physiological and behavioral responses to stress sources (Tanida et al., 2006). This provides evidence for the association between tactile stimulation and physiological recovery. Different senses convert information from the real world into electrical signals that the brain can process, forming human cognition and experiences of things (Lin, 2004). There are complex connections between neurons and brain signals triggered by sensory stimuli (Kording et al., 2007; Schreuder et al., 2016). The variations in physiological recovery effects under different sensory stimuli combinations may be related to deep-level neuronal and brain signal feedback (Gunes and Pantic, 2010; Talsma et al., 2010). This study holds significant importance in advancing research on virtual multisensory stimulation in UGSs and physiological recovery.

### 4.2 Simulated multisensory stimulation and psychological restoration

The results indicate that, compared to baseline VA stimulation (T1), the introduction of individual olfactory stimuli (T2), tactile stimuli (T3), or gustatory stimuli (T4) did not enhance emotional recovery; in fact, the recovery effects were even lower than those in the T1 group. However, these findings do not conflict with those of previous studies (Viollon et al., 2002; Zhao et al., 2018; Hedblom et al., 2019; Deng et al., 2020), as all eight sensory stimuli combinations had a restorative effect on mental health. The emphasis of this discussion lies in comparing the stronger restorative effects. Although VAOT (T5) stimulation did not significantly improve psychological recovery, stimulation with combinations of four or more types of sensory stimuli resulted in greater psychological recovery. Considering PM, NM, and the TMD score, the optimal sensory stimulation for psychological recovery is VAOG stimulation (T6). VATOG stimulation (T8) follows closely. Overall, these findings demonstrated the need for additional sensory stimulation to promote the psychological recovery effect. In the field of psychological therapy, scholars have found that single sensory stimulation may exacerbate emotional disorders, but multisensory stimulation can play a positive role in emotional regulation, serving as an adjunctive treatment for depression (Haze et al., 2002). This finding is consistent with the patterns observed in the present study.

The increase in PM and decrease in NM in the VATG group (T7) were greater than those in both the VAT group (T3) and the VAG group (T4). This finding suggests that, based on VA stimulation, gustatory-tactile stimuli interactions can enhance psychological recovery. Although research on the relationship between UGS stimuli and health recovery rarely includes the sense of touch, the association between touch and mental health has been confirmed by various studies, supporting the scientific validity of this study. Previous research has shown that C-Tactile afferents in human hairy skin are associated with the encoding of emotional information (Vallbo et al., 1999; Wessberg et al., 2003). Many researchers have referred to the pleasurable tactile sensations caused by the targeted stimulation of C-Tactile afferents as “affective touch” (Gordon et al., 2013; McGlone et al., 2014; Perini et al., 2015). Affective touch can provide information about the external world and our internal states, shaping perceptions of others and ourselves. Therefore, despite the sense of touch occurring on the external surface of the body, it is considered an interoceptive experience (Craig, 2002). During the COVID-19 pandemic, a study revealed that 60% of respondents reported experiencing varying degrees of tactile deprivation, and the degree of tactile deprivation was negatively correlated with mental health (Field et al., 2020).

Olfaction-gustatory stimuli interaction led to the greatest increase in psychological health recovery. Among the eight experimental groups, only the T6 and T8 groups were exposed to olfaction-gustation stimuli synchronously, and these two groups were the top two among all the experimental groups in terms of recovery scores. Therefore, olfaction–gustatory stimuli interactions triggered by VA basic sensory stimulation in virtual UGS environment are crucial factors for enhancing psychological recovery. Combining the results of the two aspects mentioned above, while gustation alone may not significantly increase psychological recovery on the basis of VA stimulation, the combination of olfaction or somatosensation with taste stimulation enhances psychological recovery. This emphasizes the crucial role of taste stimulation in the restorative effects of virtual UGS environment on both physical and mental health. Although gustation has rarely been explored in studies on the relationship between UGS stimuli and health recovery, there is scientific evidence supporting its high correlation with mental health. There is a significant relationship between food and emotional choices, and reducing taste input can exacerbate emotional dysfunction (Yin et al., 2021). Taste loss is closely related to emotional distress, and taste dysfunction is positively correlated with anxiety and depression (Dudine et al., 2021). Reduced taste perception can significantly predict depression and may be considered a screening indicator for depression within the diagnostic and therapeutic system (Hur et al., 2018).

### 4.3 Application of multi-sensory stimulation in virtual urban green space environment

The results show that the sensory interaction of olfactory-tactile and tactile-gustatory stimuli based on VA information can increase the antihypertensive effect, indicating that touch is an important feature for improving physiological recovery. The elderly are a group with generally high blood pressure and have problems such as mobility difficulties (Hur et al., 2018). In the areas where the elderly are concentrated in hospitals and nursing homes, Videos of the urban green environment can be played in the ward or rehabilitation room, and the plants with good natural touch (Subhashree and Saud Bijit, 2017) can be guided to better achieve the effect of assisting in lowering blood pressure. Pay attention to avoiding the trunk or leaf surface of the hair and thorns to avoid causing damage. In order to obtain better recovery effect, we should also pay attention to the activation of smell or taste, and arrange fragrant plants with pleasant smell in the room (such as *Osmanthus fragrans*, *Gardenia jasminoides*, and *Lavandula*) (Subhashree and Saud Bijit, 2017). During the viewing process, fruits can be appropriately used to activate taste, so as to achieve the best physiological recovery effect.

Smell and touch should be combined with taste stimulation to further enhance psychological recovery benefits, indicating that taste stimulation plays a key role in the effect of virtual urban green space environment on physical and mental health recovery. In special places such as the supervision institute, studies have confirmed that watching virtual videos of natural environment can relieve the anxiety and reduce the pressure of the supervised personnel (Nadkarni et al., 2021). The results of this study are the same, and further prove that when receiving the stimulation of natural environment video, synchronizing with the activation of smell and taste can better obtain psychological recovery. Previous studies have shown that watching videos of natural environment during recess can help students recover their attention (Luo et al., 2023). The results of this study further indicate that during recess or some activity classes at school, reasonable interspersed videos of virtual natural environment should be played while guiding students to eat fruits (such as loquat, peach, pear, etc.). In addition, potted plants with natural fragrance are used in the classroom to activate students’ sense of smell, which can better help students relieve mental pressure and complete the next class better.

### 4.4 Limitations and future studies

The main limitation of this study is that ecological validity was not assessed. Despite the widespread use of simulated methods involving images and sounds (Cañas et al., 2009; Wang et al., 2017; Deng et al., 2020), this study delved into the rarely explored realms of tactile and gustatory stimuli for virtual multisensory stimulation in UGSs. In contrast to the more established methods used in visual, auditory, and olfactory simulation research (Song et al., 2019; Deng et al., 2020; Ratcliffe, 2021), there has been minimal scholarly comparison and validation of methods simulating touch and taste. Therefore, compared to stimuli generated in real-world environments, there may be instances where simulated stimuli do not entirely replicate real-world experiences, potentially impacting the results to a certain extent. Therefore, the conclusion of this study is aimed at the restoration effect of virtual urban green space environment, and has not been applied to the planning and design of real environment.

Future research could explore the following three aspects. First, to minimize the potential influence of demographic variables on the results, this study controlled for participants’ social attributes by recruiting university students of similar age. However, future research could broaden participant demographics, including individuals from various age groups, to conduct studies across multiple age ranges. Second, we can learn from previous scholars’ evaluation methods on the ecological effects of soundscape reproduction to evaluate the ecological effects of the multi-sensory stimulation method used in this experiment (Tarlao et al., 2022), so that the research conclusions can be applied to the planning and design of more realistic scenes. Third, touch and taste are aspects that have rarely been investigated in research on the relationship between sensory stimuli in UGSs and psychophysical recovery. Although this study included touch and taste, the subcategories were not refined. Therefore, future research can refine the different types of touch and taste stimuli and further deepen the related research.

## 5 Conclusion

In terms of physiological recovery, the blood pressure of the 8 experimental groups decreased significantly after the experiment, indicating that the virtual urban green space environment has a certain recovery effect on physiological state, and could effectively relieve the public’s stress state. While the combination of VAOTG stimuli in the multisensory group resulted in the best blood pressure recovery (*p* < 0.05), there was a non-linear relationship between blood pressure recovery and the number of sensory activations. Tactile is an important sense to enhance the physiological recovery effect. Within the context of VA stimuli, the interaction of olfactory-tactile and tactile-gustatory stimuli enhanced physiological recovery.

In terms of psychological recovery, the common trigger of olfactory-gustatory is the most key element to enhance psychological recovery through multi-sensory stimulation of virtual urban green space environment. VAOG stimulation had the best effect on psychological recovery (*p* < 0.05), followed by VAOTG stimulation (*p* < 0.05). Gustatory is an important sense to enhance the psychological recovery effect, and both the tactile-gustatory interaction and the olfactory-gustatory interaction significantly enhance the recovery effect. At the same time, the psychological recovery effect obtained by four or more sensory combinations was higher than that obtained by two or three sensory stimulation groups, which explained the necessity of multiple sensory activation on psychological recovery effect to a certain extent.

## Data availability statement

The datasets presented in this article are not readily available because we do not provide public access to the dataset due to protection of the privacy of the participants. Regarding the details of the data, please contact the corresponding author. Requests to access the datasets should be directed to XL, lixi@sicau.edu.cn.

## Ethics statement

The study was performed with the approval of the local Ethics Committee of the College of Landscape Architecture, Sichuan Agricultural University, China. The studies were conducted in accordance with the local legislation and institutional requirements. The participants provided their written informed consent to participate in this study. Written informed consent was obtained from the individual(s) for the publication of any identifiable images or data included in this article.

## Author contributions

CS: Visualization, Validation, Software, Methodology, Investigation, Formal analysis, Data curation, Conceptualization, Writing – review & editing, Writing – original draft. SC: Investigation, Conceptualization, Writing – review & editing, Writing – original draft. HL: Writing – review & editing, Data curation. YH: Writing – review & editing, Data curation. SJ: Writing – review & editing, Data curation. BG: Writing – review & editing, Investigation. NL: Writing – review & editing, Investigation. KL: Writing – review & editing, Investigation. PZ: Writing – review & editing, Investigation. CZ: Writing – review & editing, Investigation. EF: Writing – review & editing, Investigation. MJ: Writing – review & editing, Investigation. XL: Writing – review & editing, Supervision, Resources, Project administration, Funding acquisition, Conceptualization.
