# Supplementary material for: Effects of simulated multi-sensory stimulation integration on physiological and psychological restoration in virtual urban green space environment
Source: Front Psychol. 2024 Jun 20;15:1382143. doi: 10.3389/fpsyg.2024.1382143 (PMC11223631; doi:10.3389/fpsyg.2024.1382143)
Supplement: Supplementary file 1 [file Table_1.DOCX]

Supplementary Material

Effects of multisensory stimulation integration in urban green spaces on physiological and psychological restoration

Chen Song^1†^, Saixin Cao^1†^, Hao Luo^2^, Yinghui Huang^1^, Siwei Jiang^1^, Baimeng Guo^1^, Nian Li^1^, Kai Li^1^, Ping Zhang^1^, Chunyan Zhu^1^, Erkang Fu^1^, Mingyan Jiang^1^, Xi Li^1^*

*** Correspondence:** Xi Li: [lixi@sicau.edu.cn](mailto:lixi@sicau.edu.cn)

**The Abbreviated Profile of Mood States (POMS)**

Below is a list of words that describe feelings people have. Please **CIRCLE THE NUMBER THAT BEST DESCRIBES** **HOW YOU FEEL RIGHT NOW**.

|  |  | Not At All | A Little | Moderately | Quite a lot | Extremely |
| --- | --- | --- | --- | --- | --- | --- |
|  | Tense | 0 | 1 | 2 | 3 | 4 |
|  | Angry | 0 | 1 | 2 | 3 | 4 |
|  | Worn Out | 0 | 1 | 2 | 3 | 4 |
|  | Unhappy | 0 | 1 | 2 | 3 | 4 |
|  | Proud | 0 | 1 | 2 | 3 | 4 |
|  | Lively | 0 | 1 | 2 | 3 | 4 |
|  | Confused | 0 | 1 | 2 | 3 | 4 |
|  | Sad | 0 | 1 | 2 | 3 | 4 |
|  | Active | 0 | 1 | 2 | 3 | 4 |
|  | On-edge | 0 | 1 | 2 | 3 | 4 |
|  | Grouchy | 0 | 1 | 2 | 3 | 4 |
|  | Ashamed | 0 | 1 | 2 | 3 | 4 |
|  | Energetic | 0 | 1 | 2 | 3 | 4 |
|  | Hopeless | 0 | 1 | 2 | 3 | 4 |
|  | Uneasy | 0 | 1 | 2 | 3 | 4 |
|  | Restless | 0 | 1 | 2 | 3 | 4 |
|  | Unable to concentrate | 0 | 1 | 2 | 3 | 4 |
|  | Fatigued | 0 | 1 | 2 | 3 | 4 |
|  | Competent | 0 | 1 | 2 | 3 | 4 |
|  | Annoyed | 0 | 1 | 2 | 3 | 4 |
|  | Discouraged | 0 | 1 | 2 | 3 | 4 |
|  | Resentful | 0 | 1 | 2 | 3 | 4 |
|  | Nervous | 0 | 1 | 2 | 3 | 4 |
|  | Miserable | 0 | 1 | 2 | 3 | 4 |
|  | Confident | 0 | 1 | 2 | 3 | 4 |
|  | Bitter | 0 | 1 | 2 | 3 | 4 |
|  | Exhausted | 0 | 1 | 2 | 3 | 4 |
|  | Anxious | 0 | 1 | 2 | 3 | 4 |
|  | Helpless | 0 | 1 | 2 | 3 | 4 |
|  | Weary | 0 | 1 | 2 | 3 | 4 |
|  | Satisfied | 0 | 1 | 2 | 3 | 4 |
|  | Bewildered | 0 | 1 | 2 | 3 | 4 |
|  | Furious | 0 | 1 | 2 | 3 | 4 |
|  | Full of Pep | 0 | 1 | 2 | 3 | 4 |
|  | Worthless | 0 | 1 | 2 | 3 | 4 |
|  | Forgetful | 0 | 1 | 2 | 3 | 4 |
|  | Vigorous | 0 | 1 | 2 | 3 | 4 |
|  | Uncertain about things | 0 | 1 | 2 | 3 | 4 |
|  | Bushed | 0 | 1 | 2 | 3 | 4 |
|  | Embarrassed | 0 | 1 | 2 | 3 | 4 |

THANK YOU FOR YOUR COOPERATION

PLEASE BE SURE YOU HAVE ANSWERED EVERY ITEM
