# Supplementary material for: Effects of simulated multi-sensory stimulation integration on physiological and psychological restoration in virtual urban green space environment
Source: Front Psychol. 2024 Jun 20;15:1382143. doi: 10.3389/fpsyg.2024.1382143 (PMC11223631; doi:10.3389/fpsyg.2024.1382143)
Supplement: Supplementary file 2 [file Presentation_1.PPTX]

## Slide 1
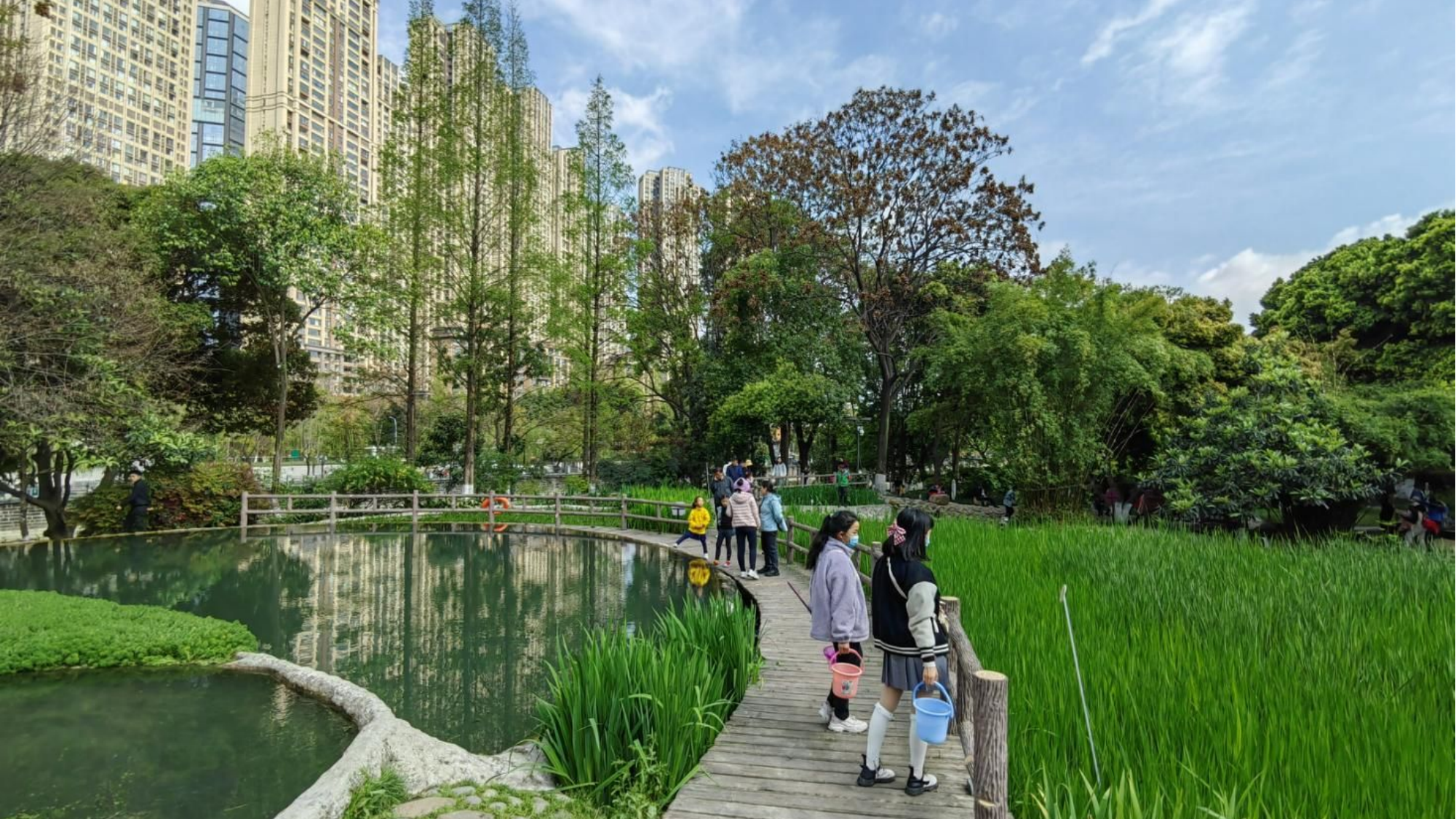

## Slide 2
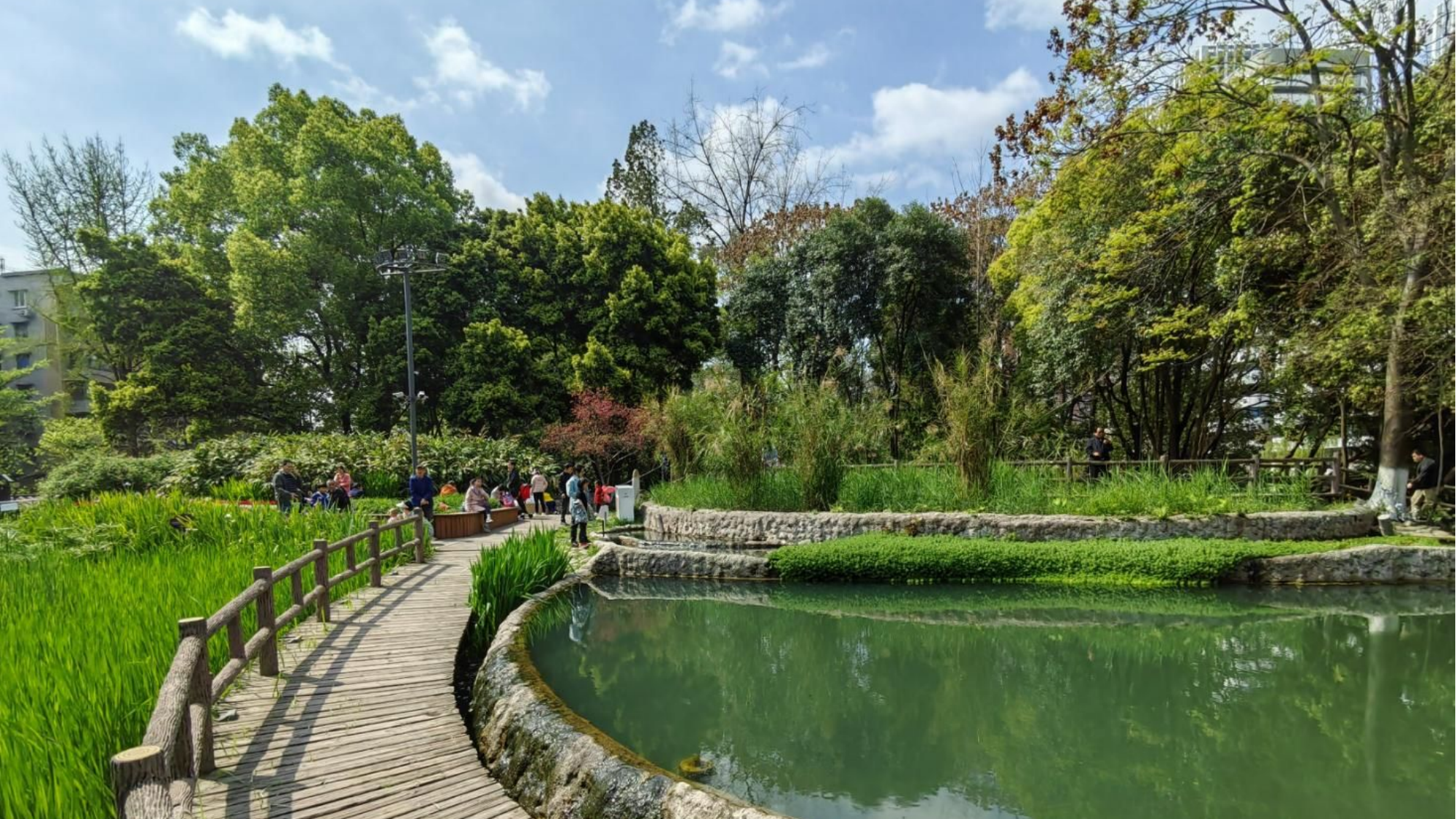

## Slide 3
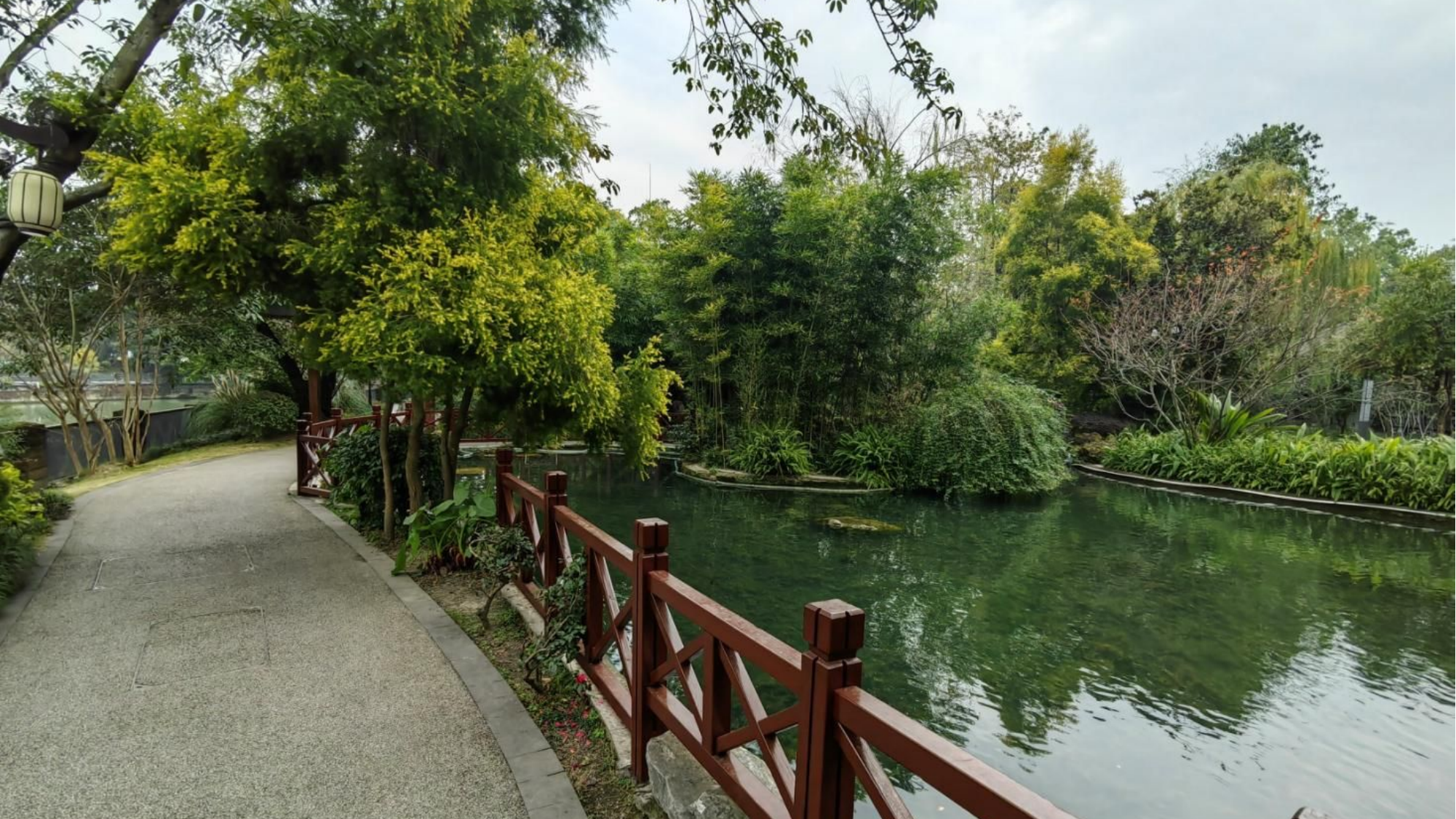

## Slide 4
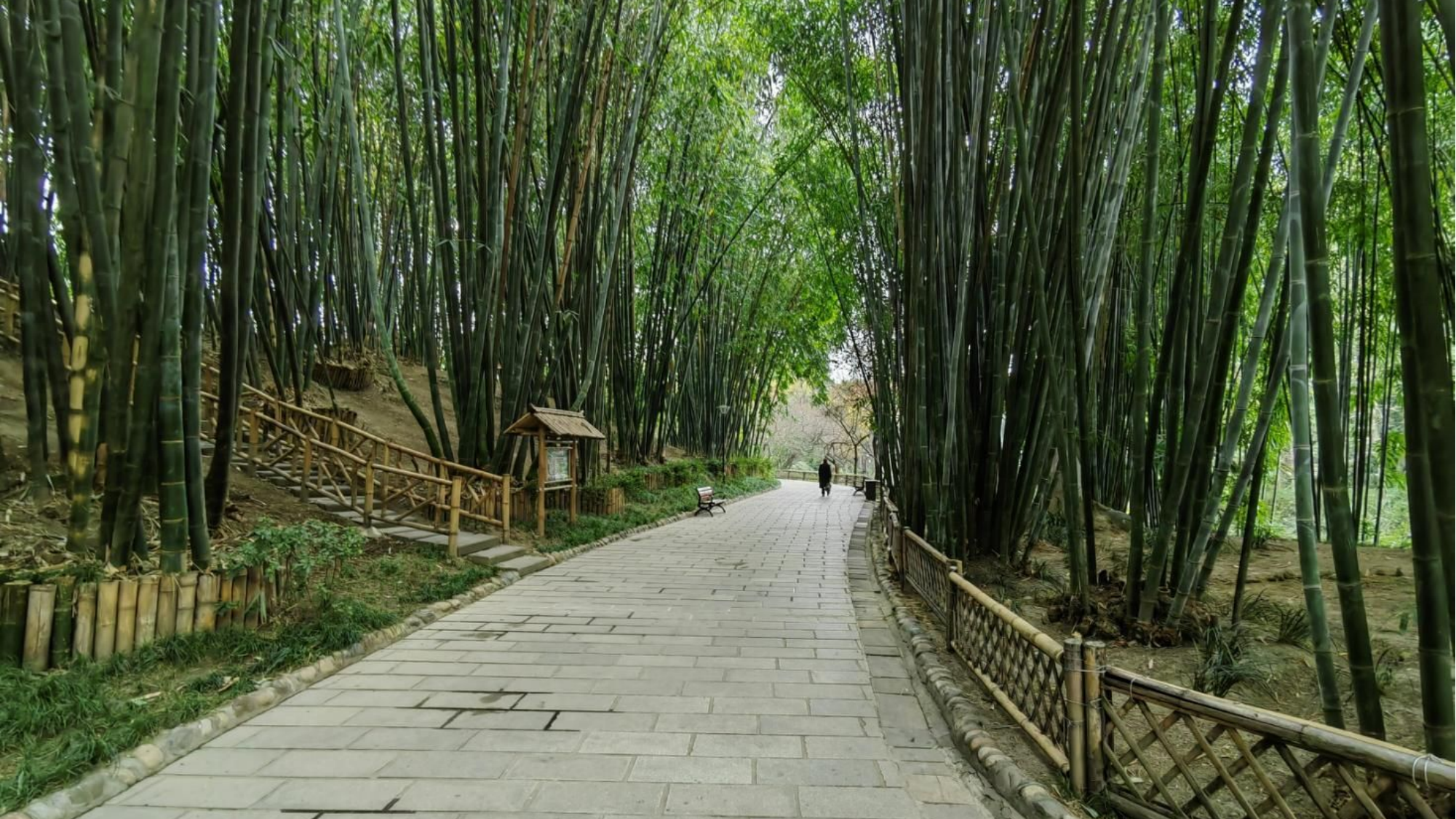

## Slide 5
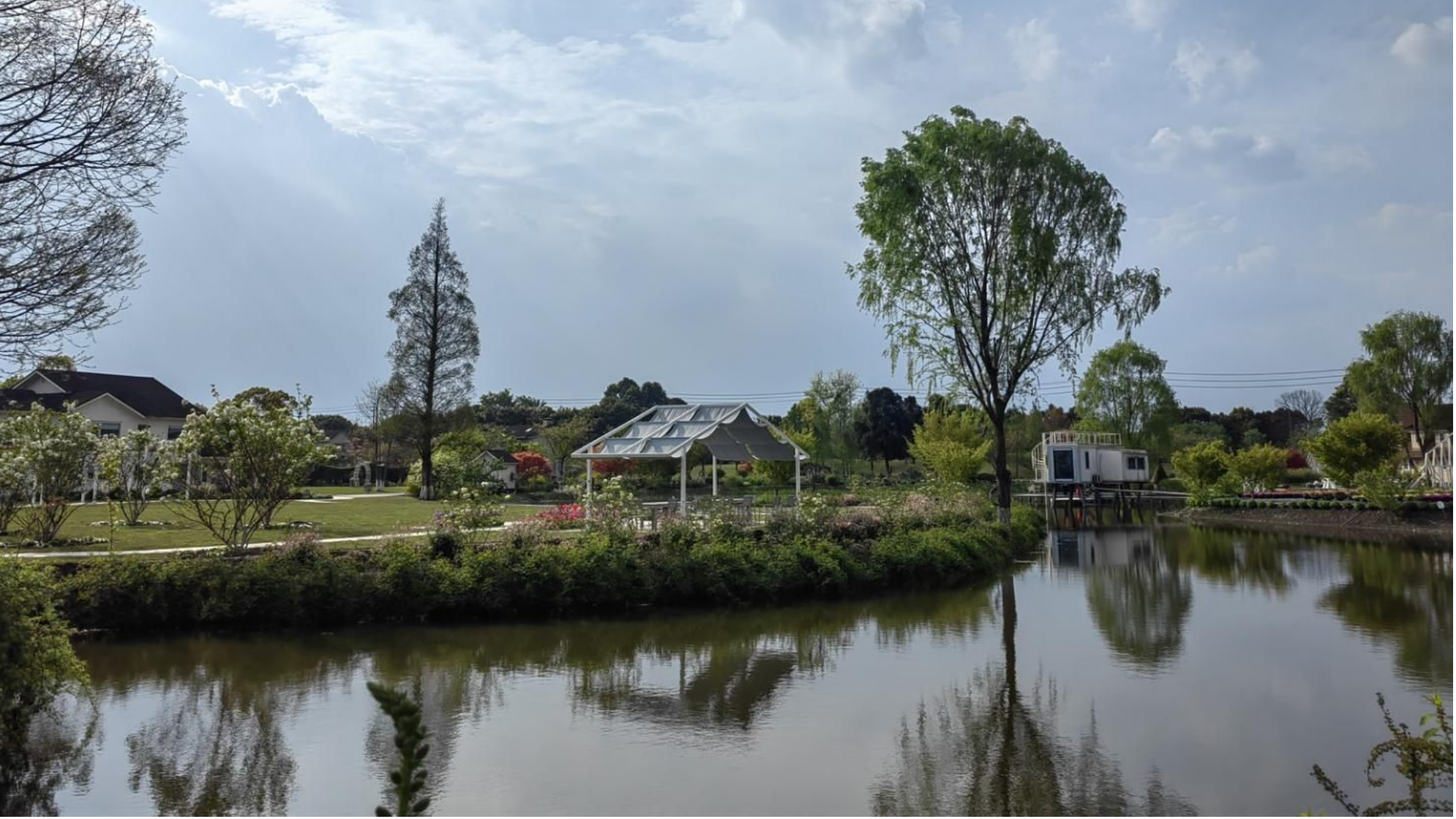

## Slide 6
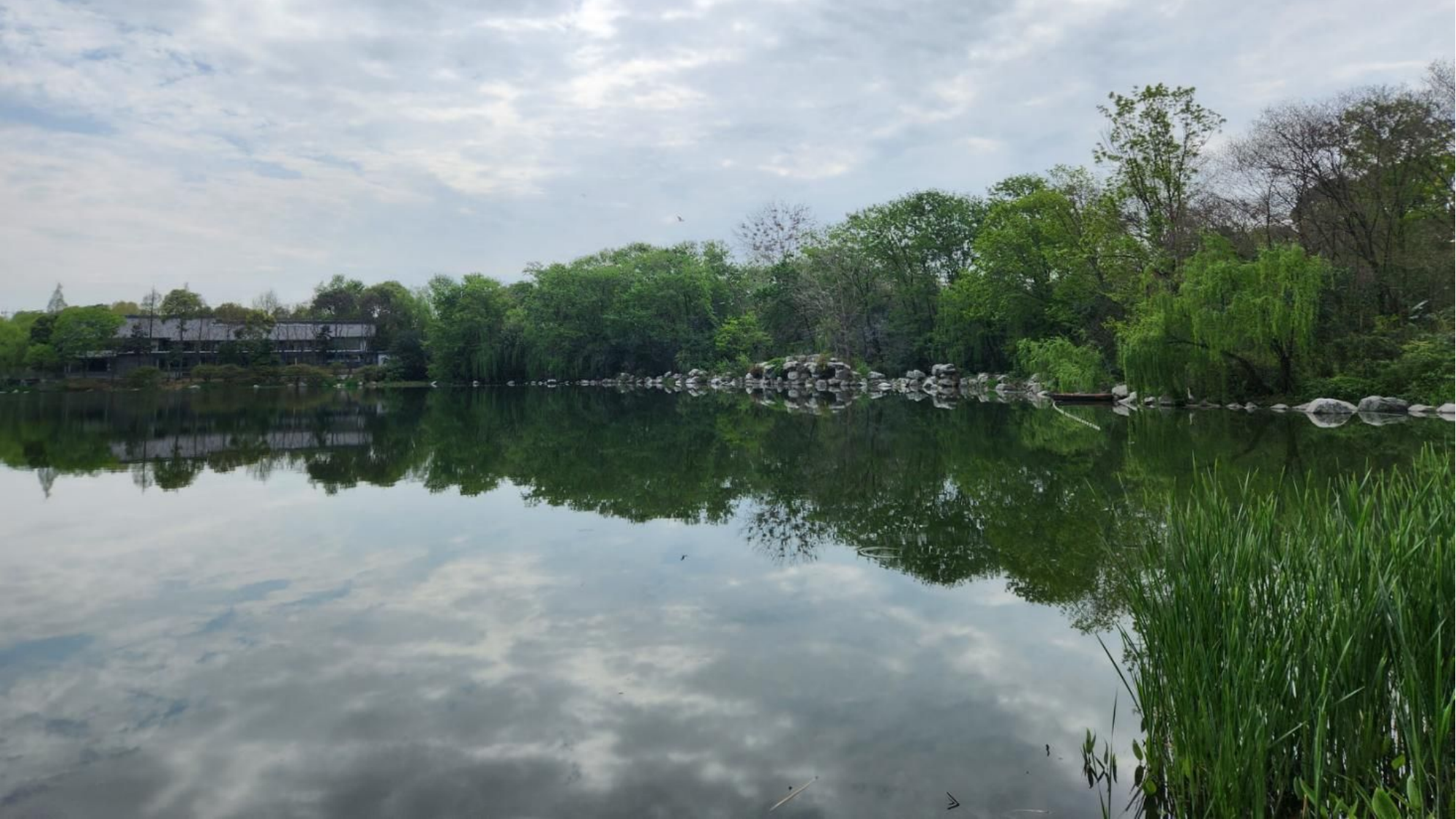

## Slide 7
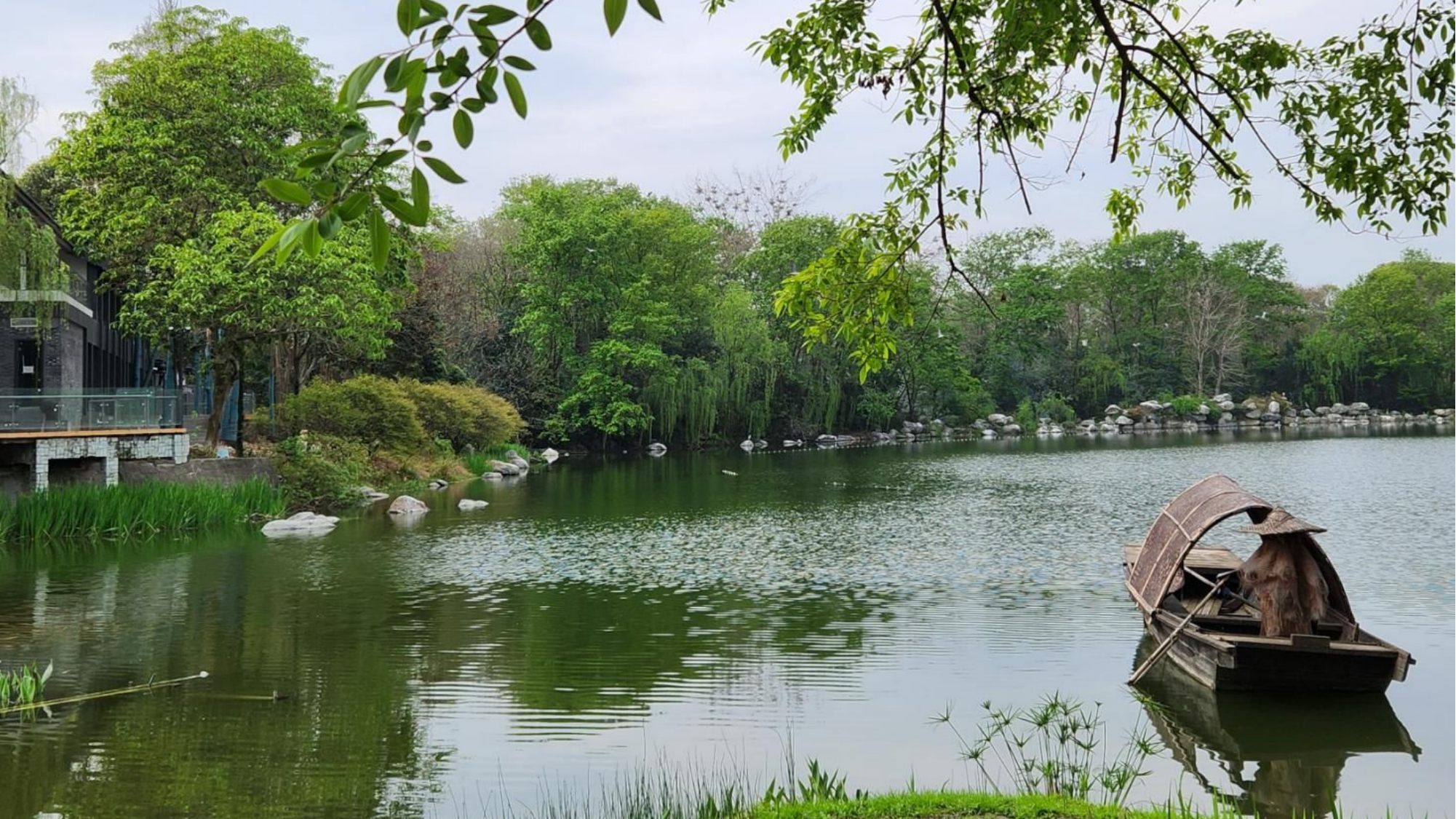

## Slide 8
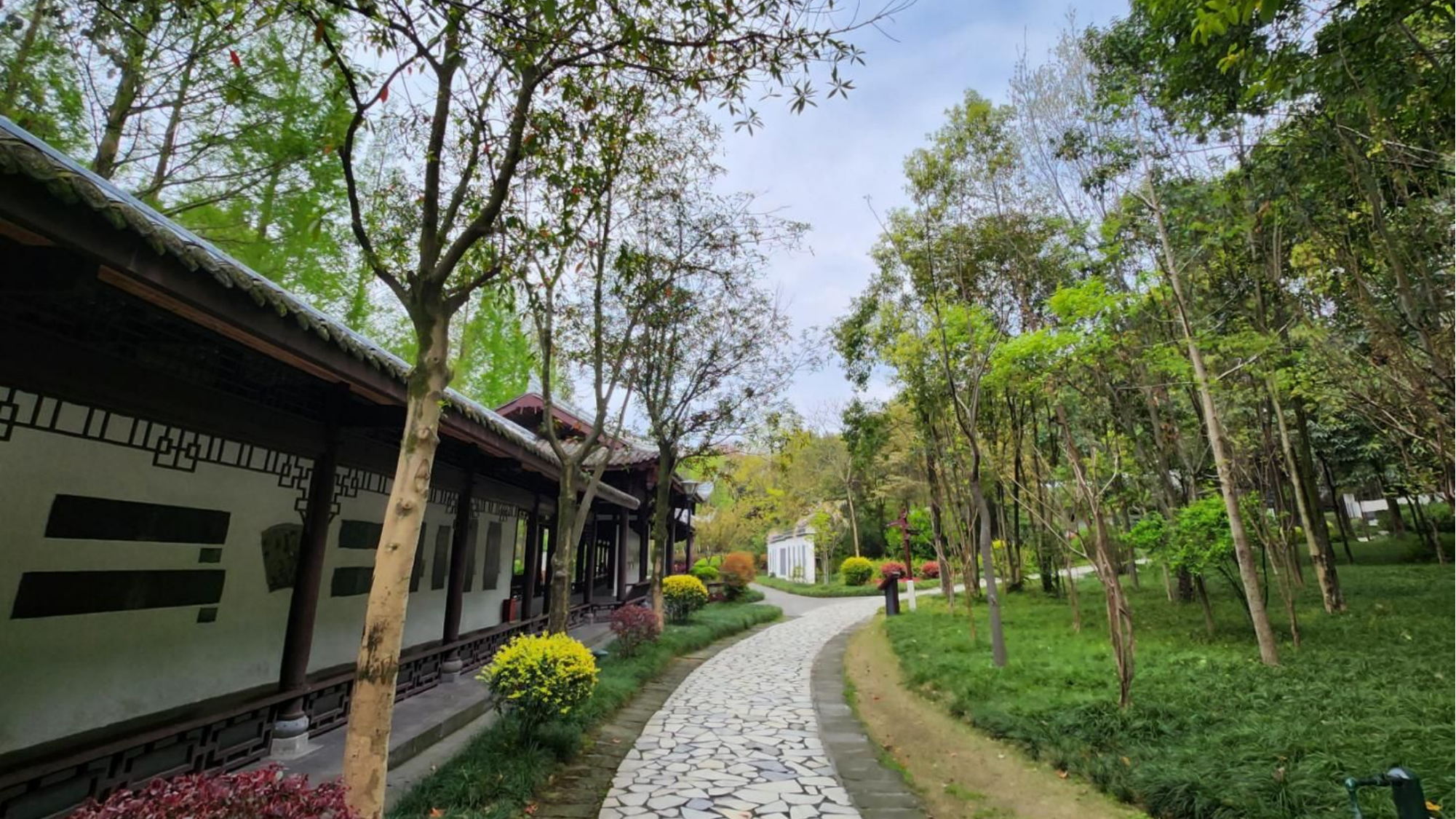

## Slide 9
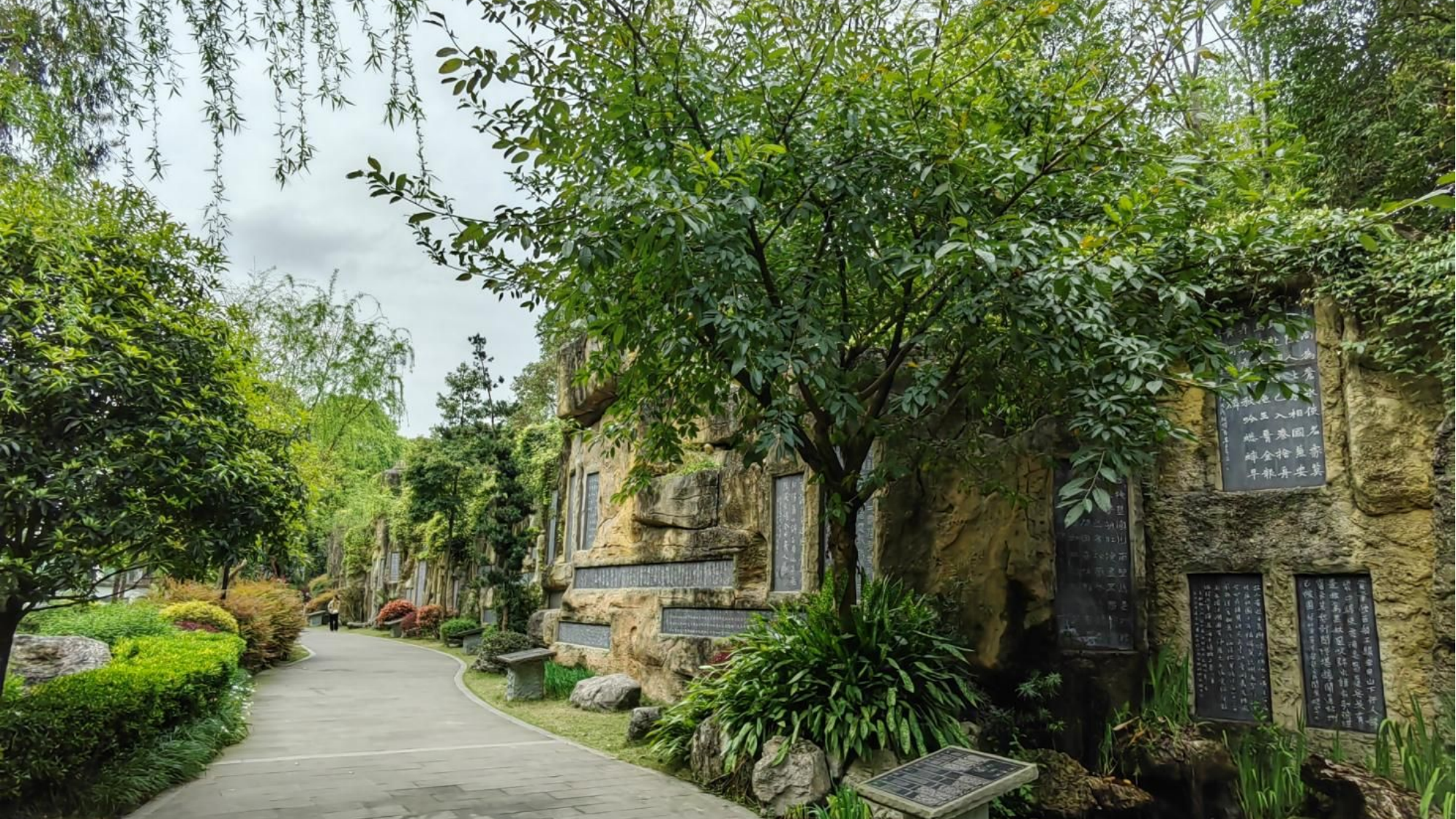

## Slide 10
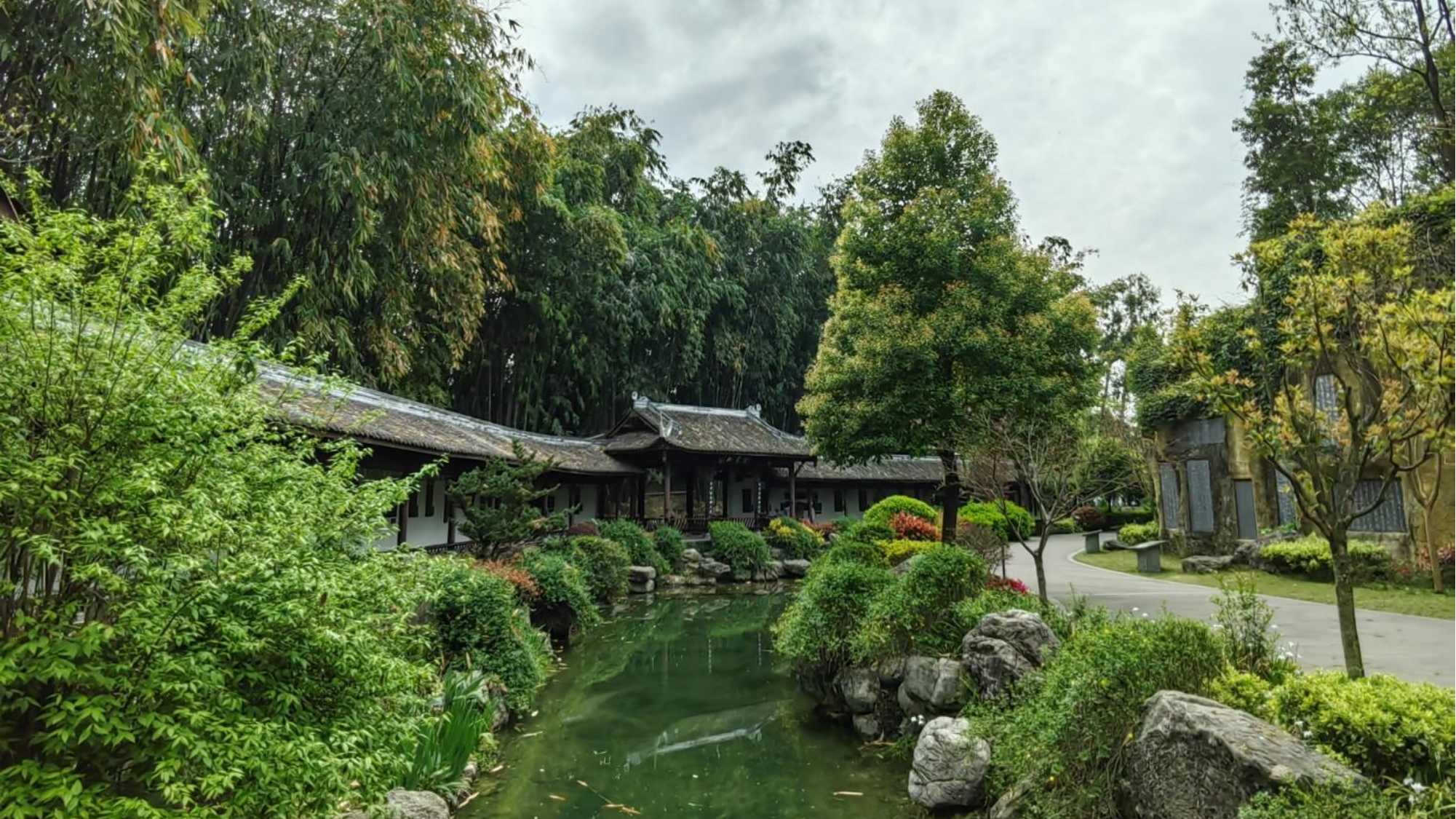

## Slide 11
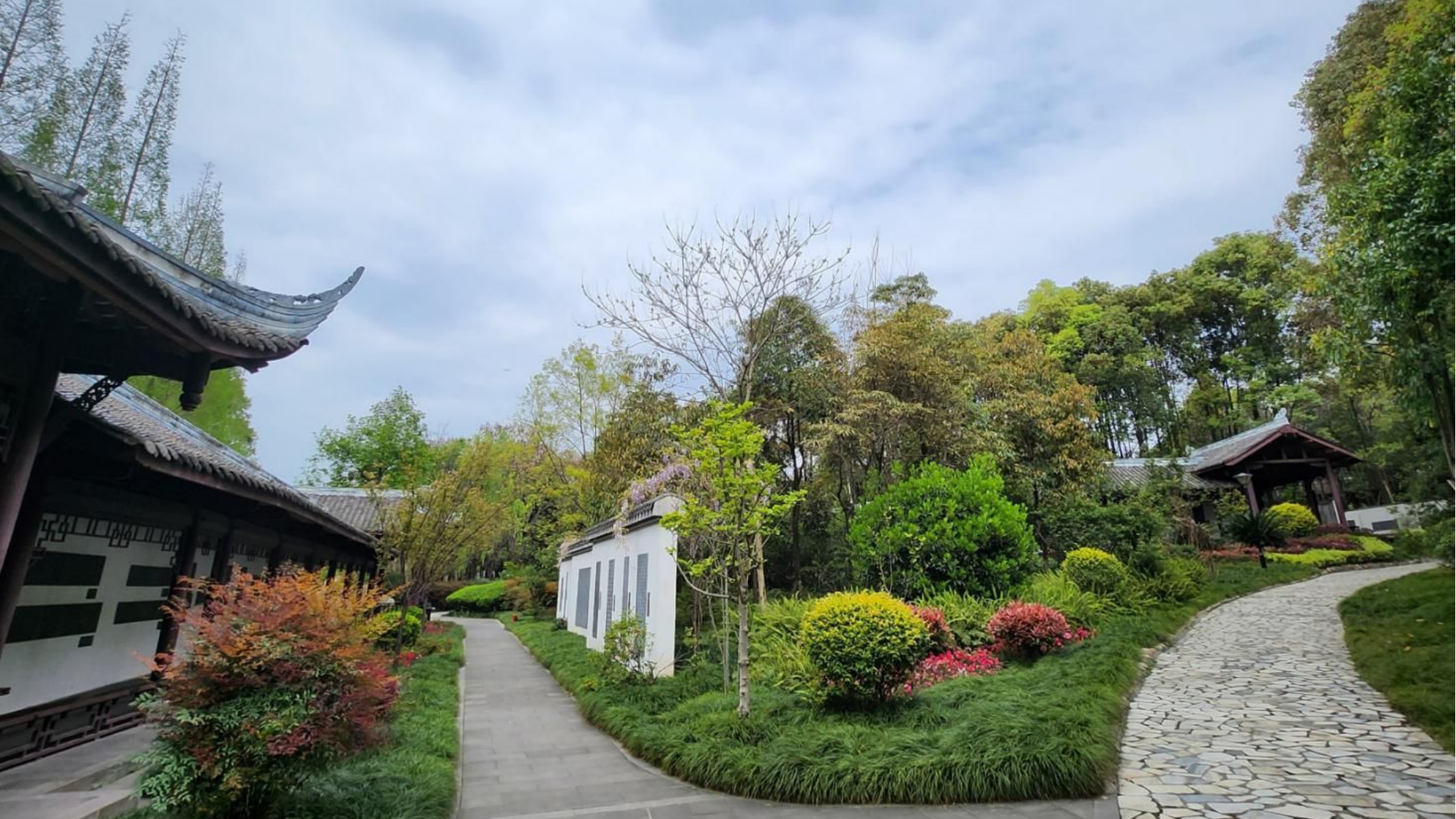

## Slide 12
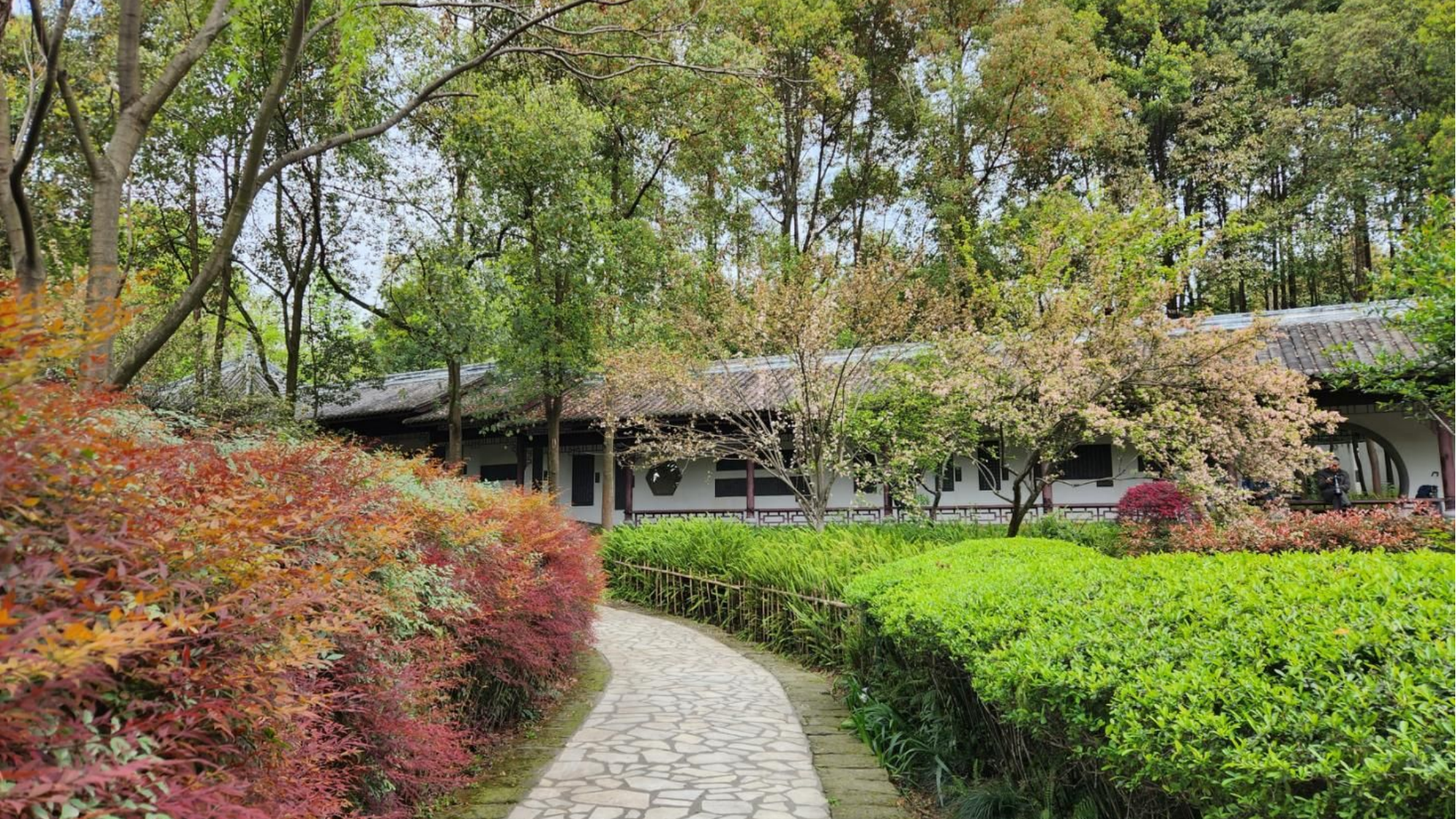

## Slide 13
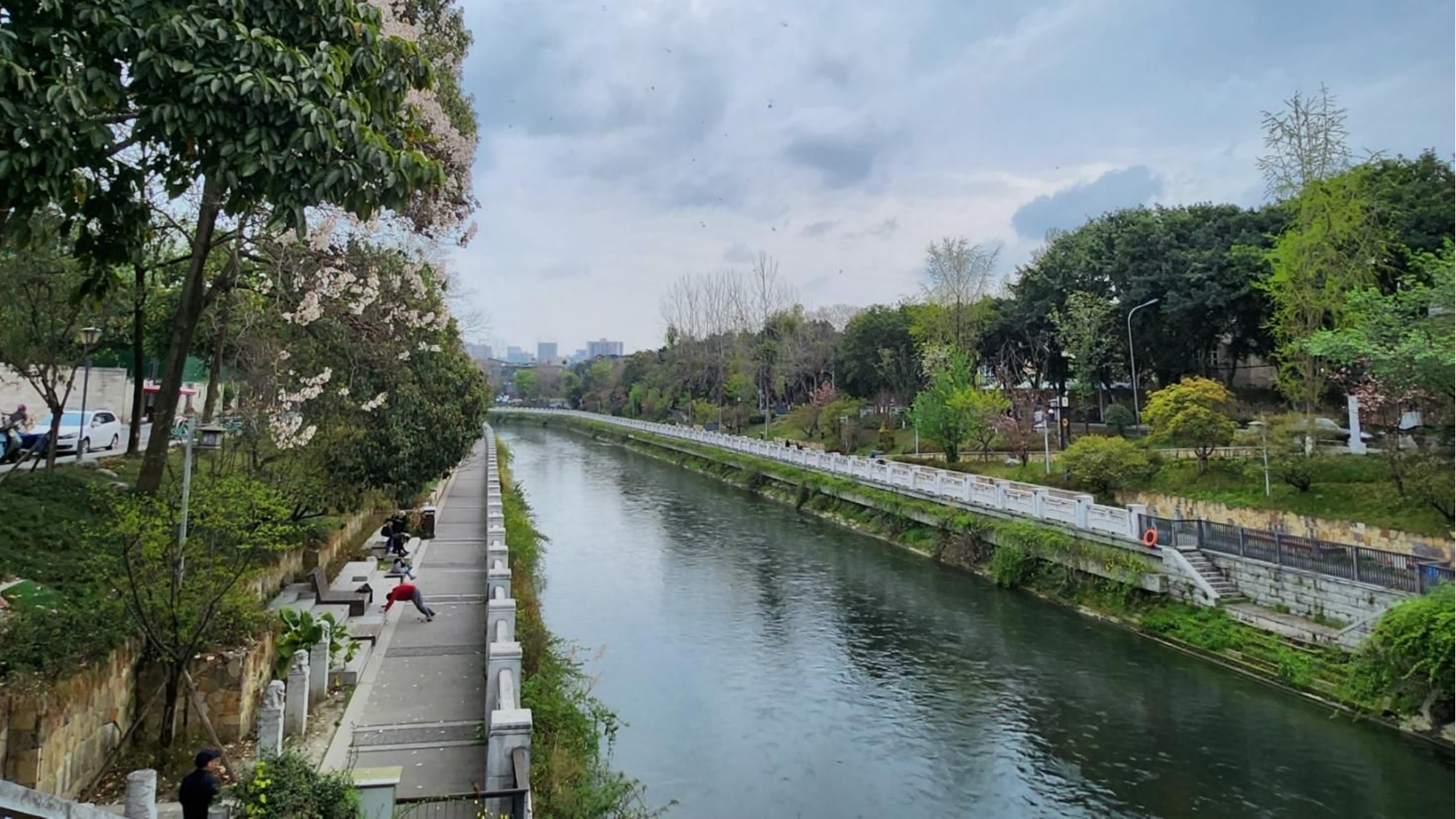

## Slide 14
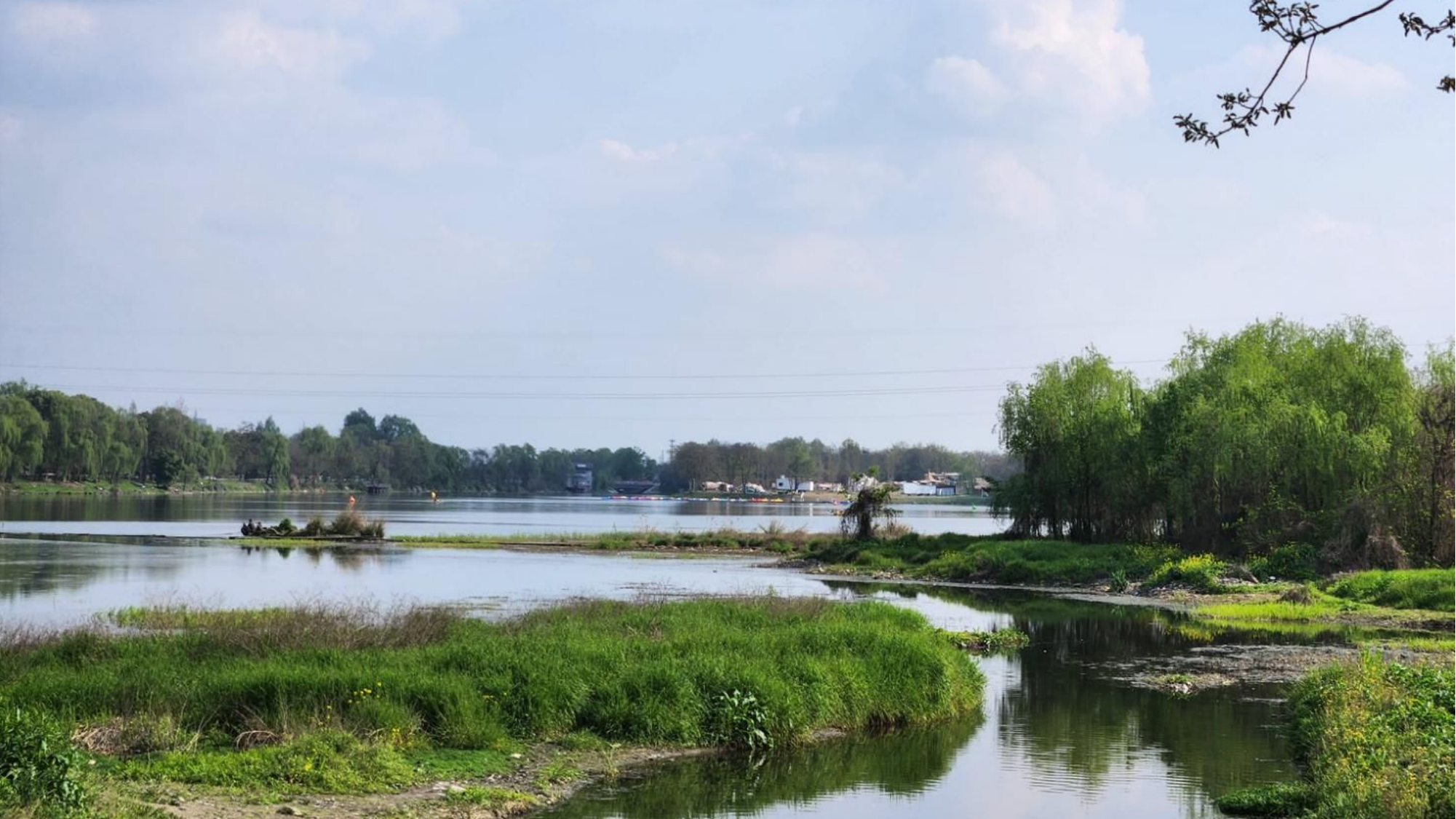

## Slide 15
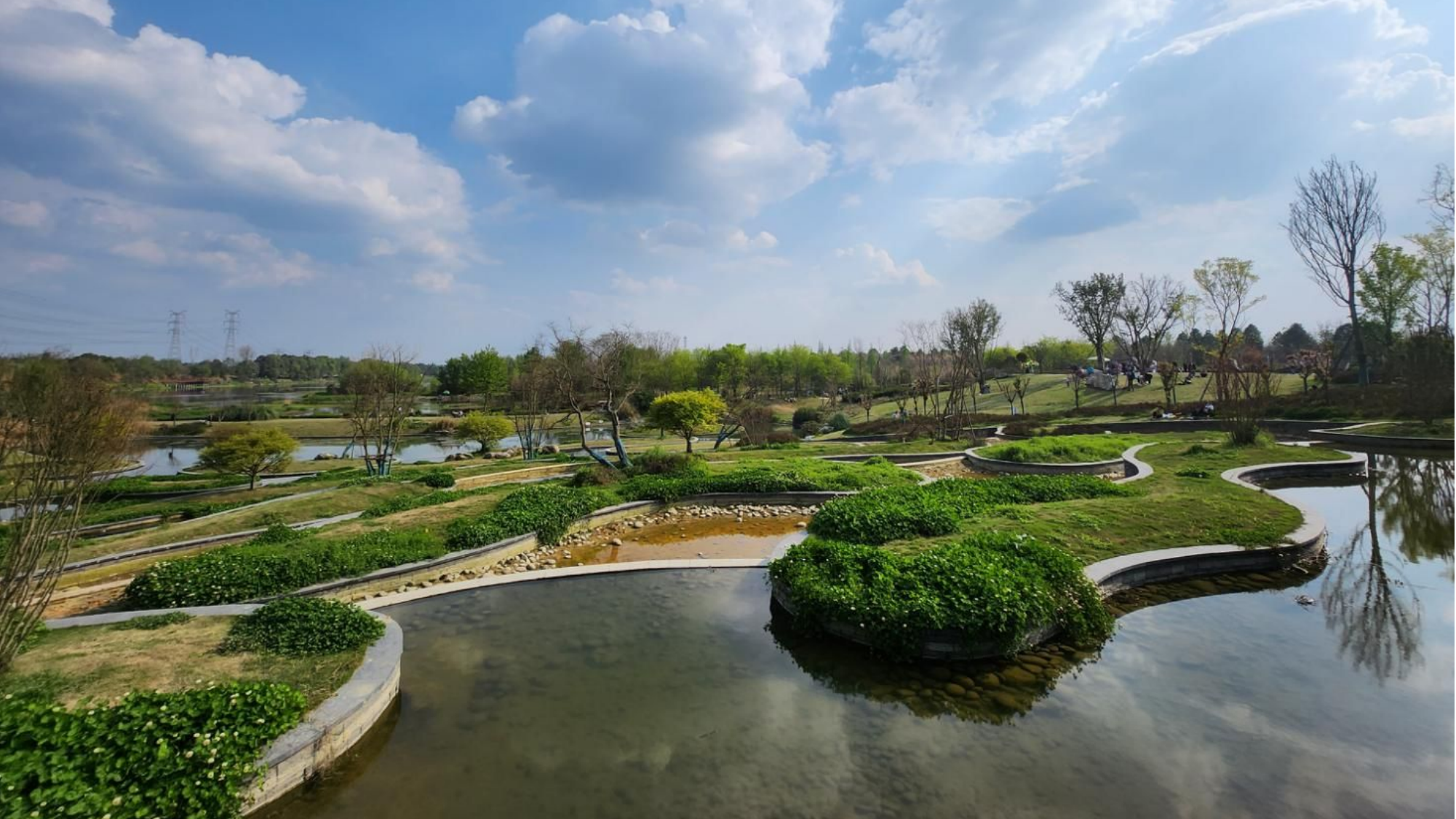

## Slide 16
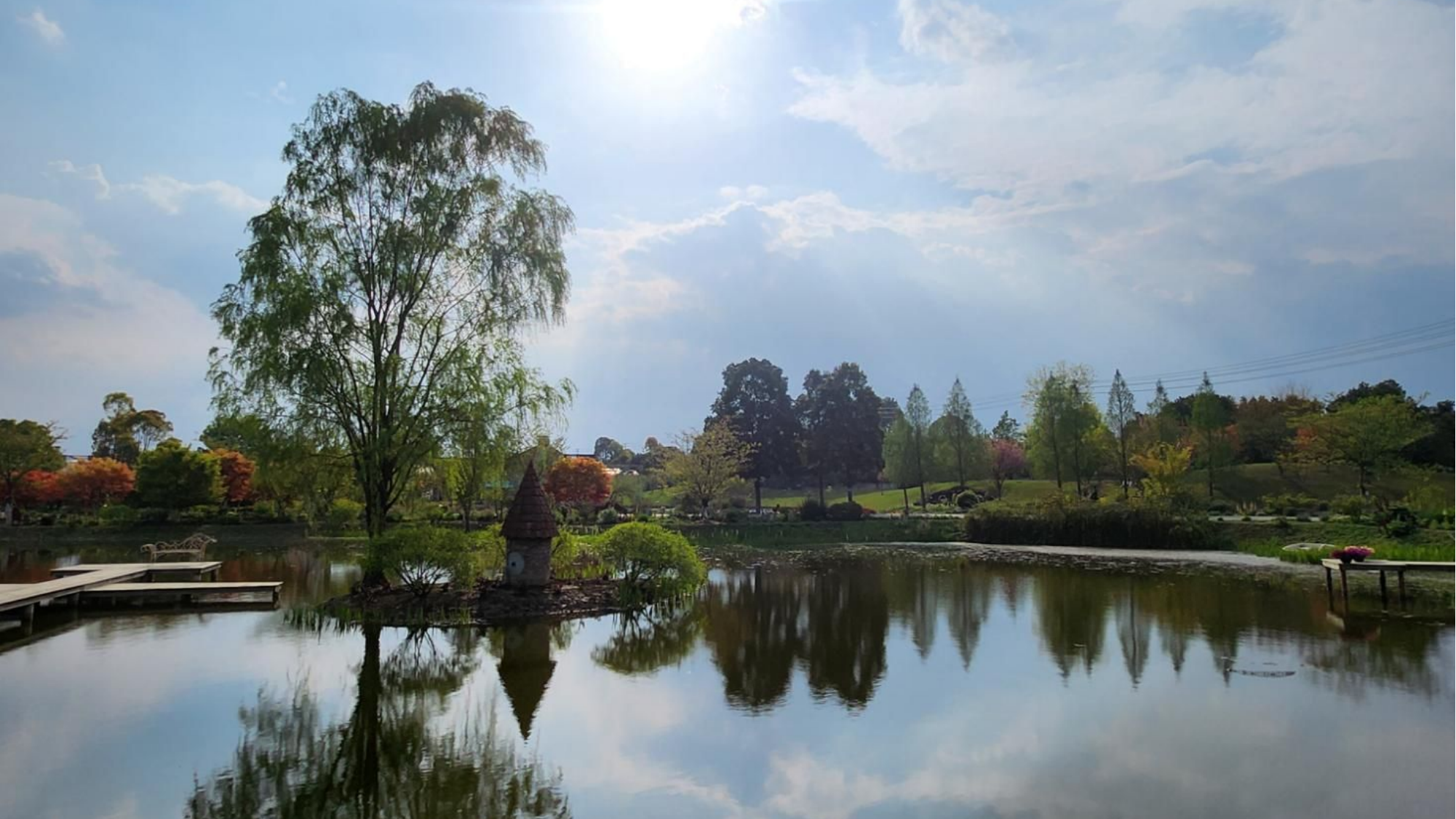

## Slide 17
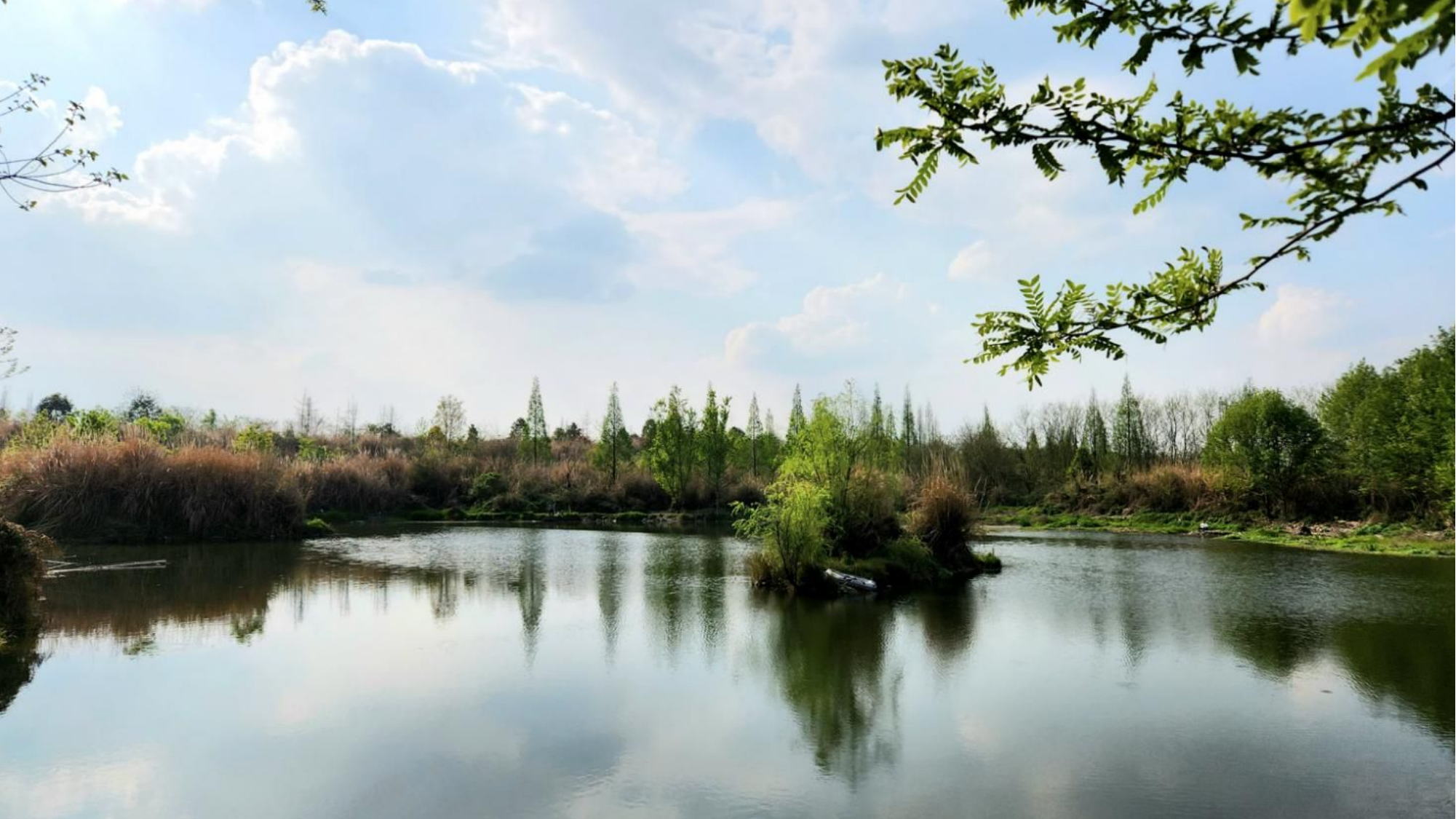

## Slide 18
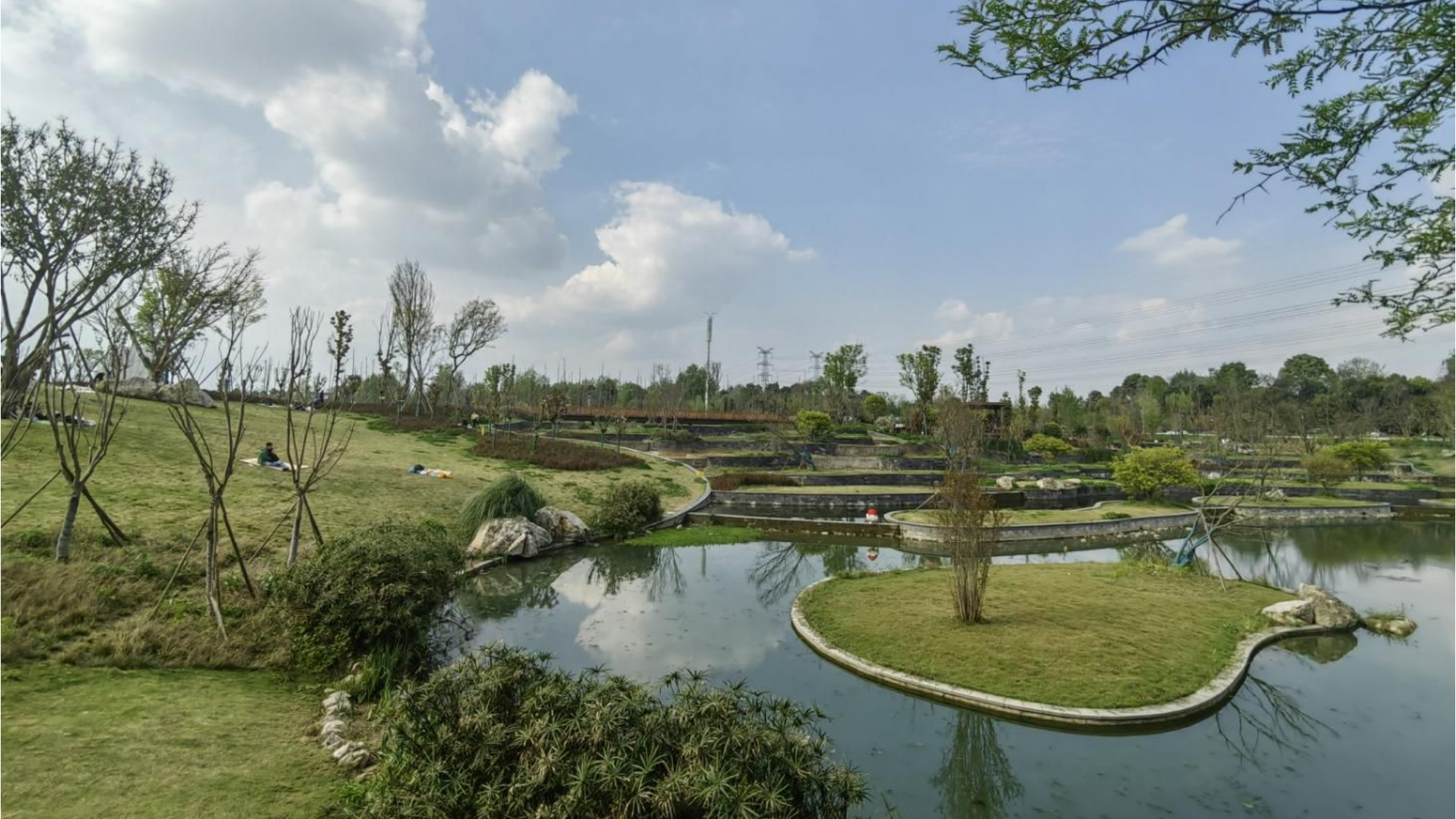

## Slide 19
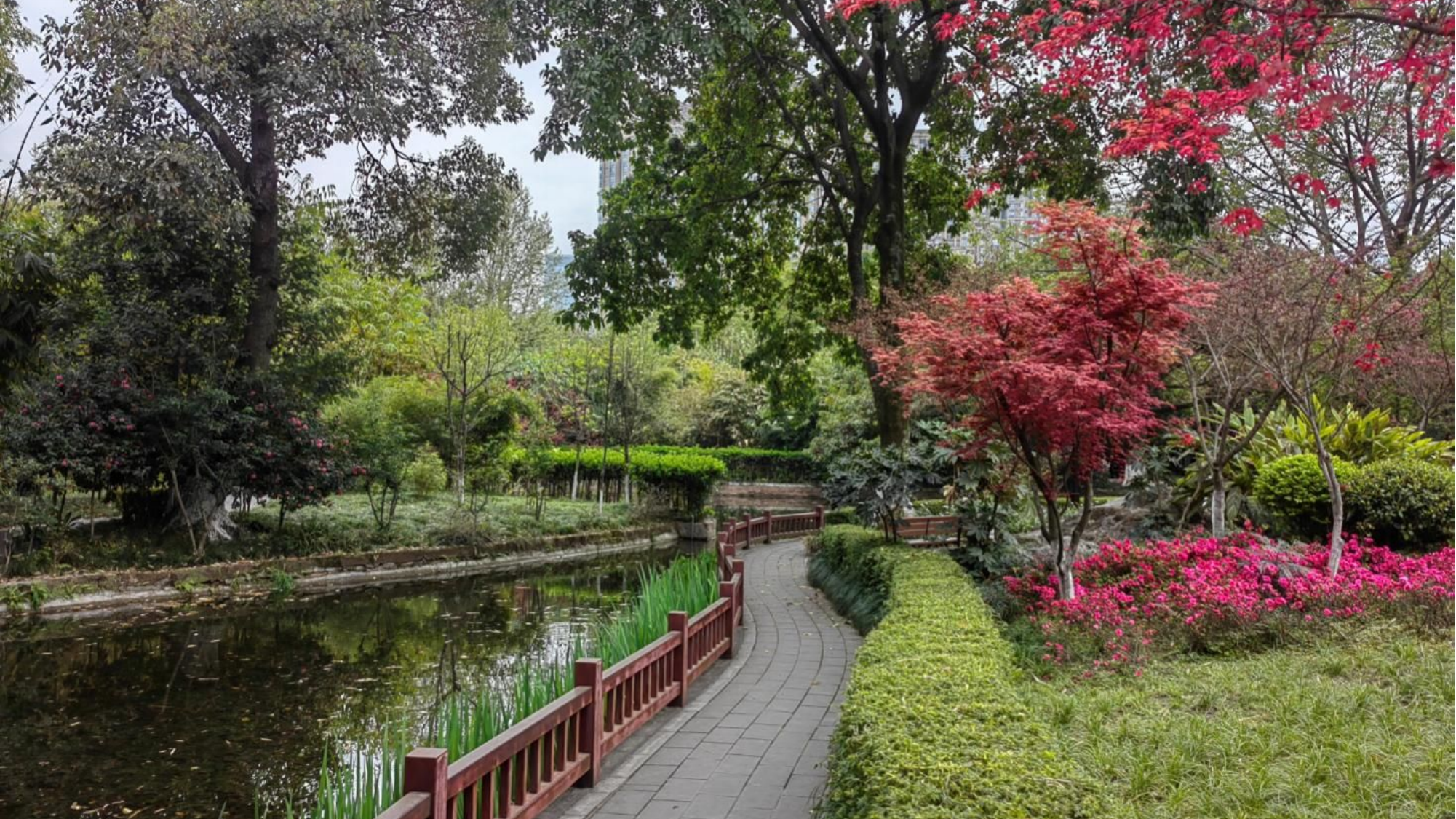

## Slide 20
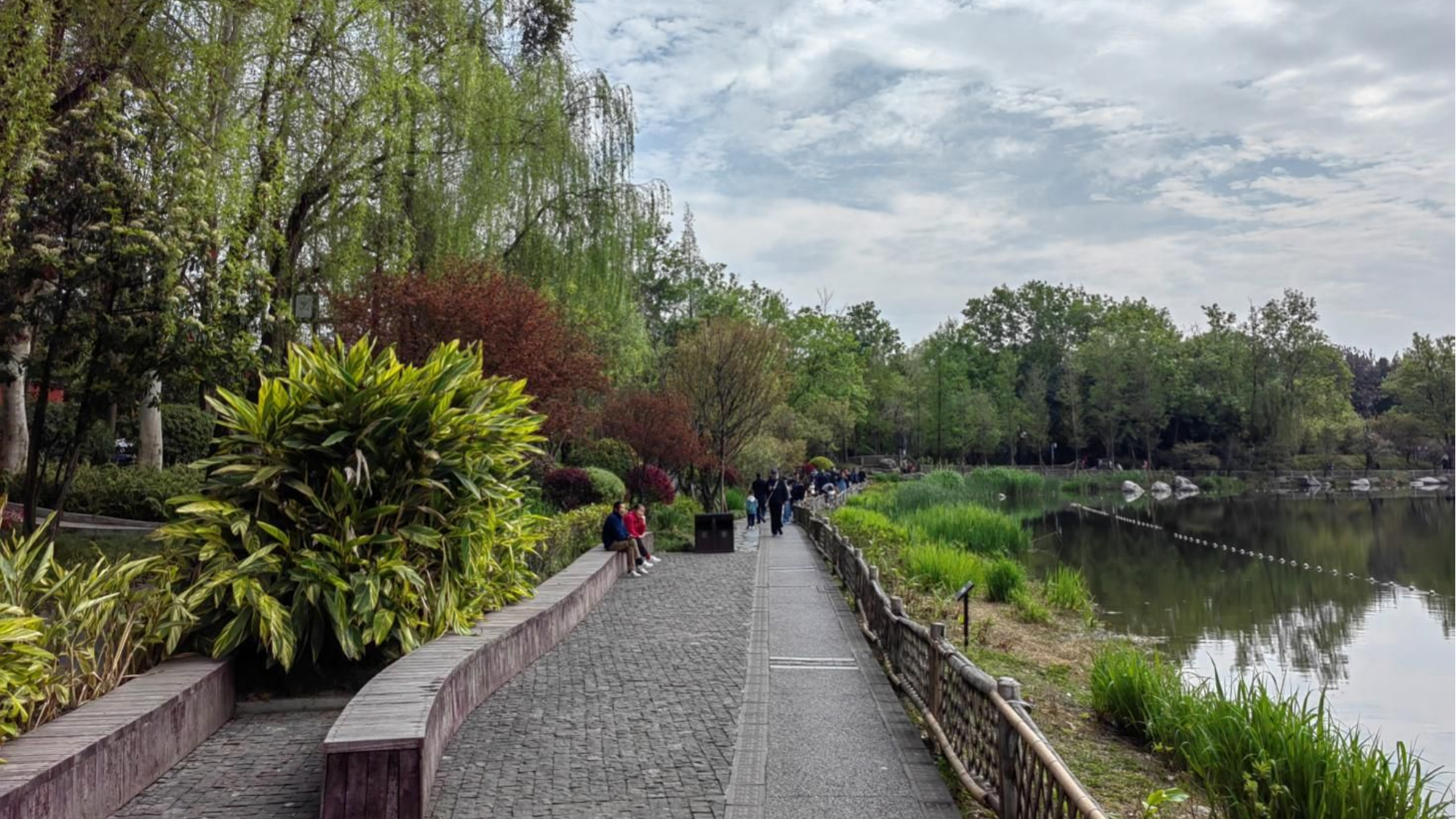

## Slide 21
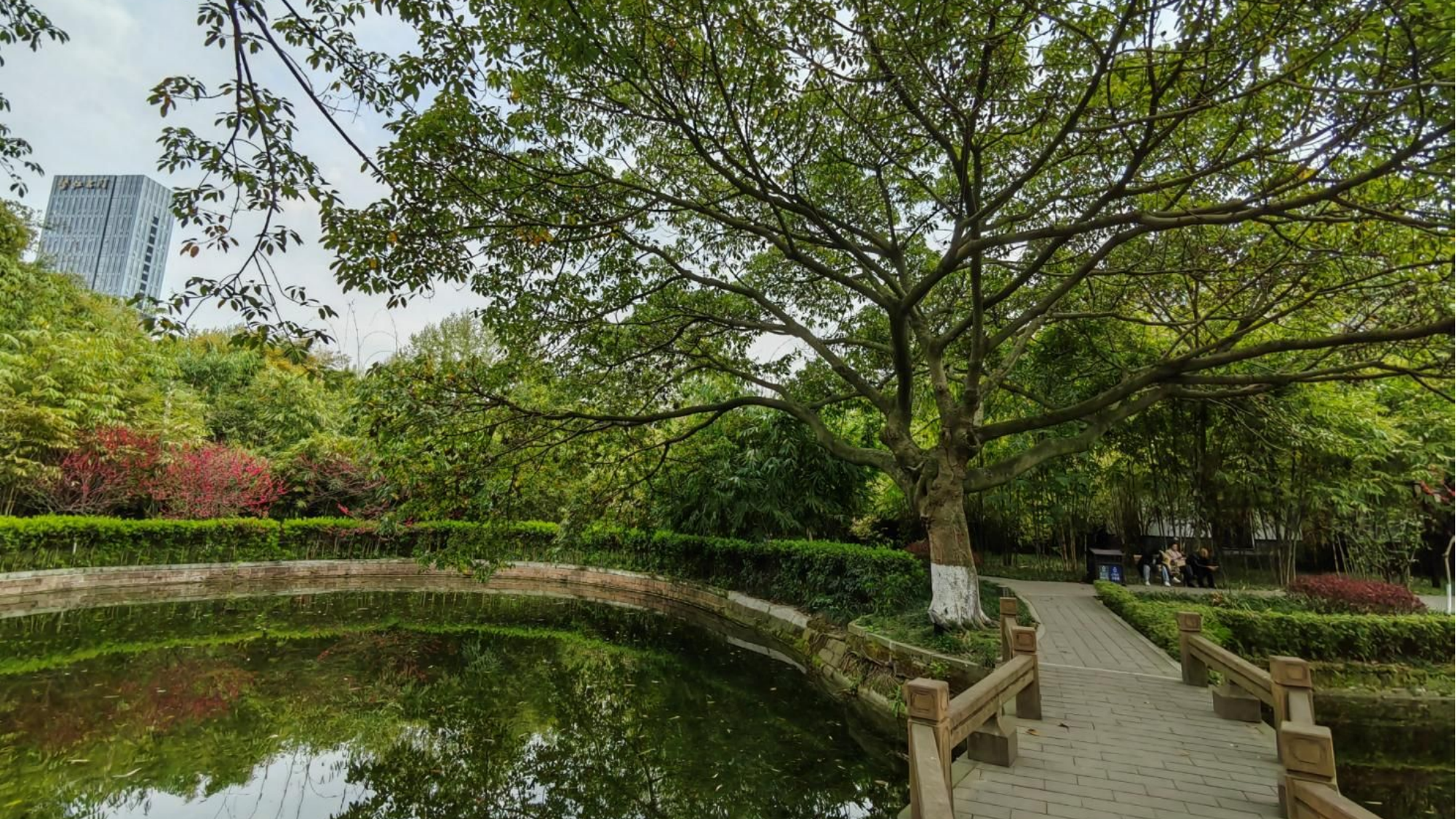

## Slide 22
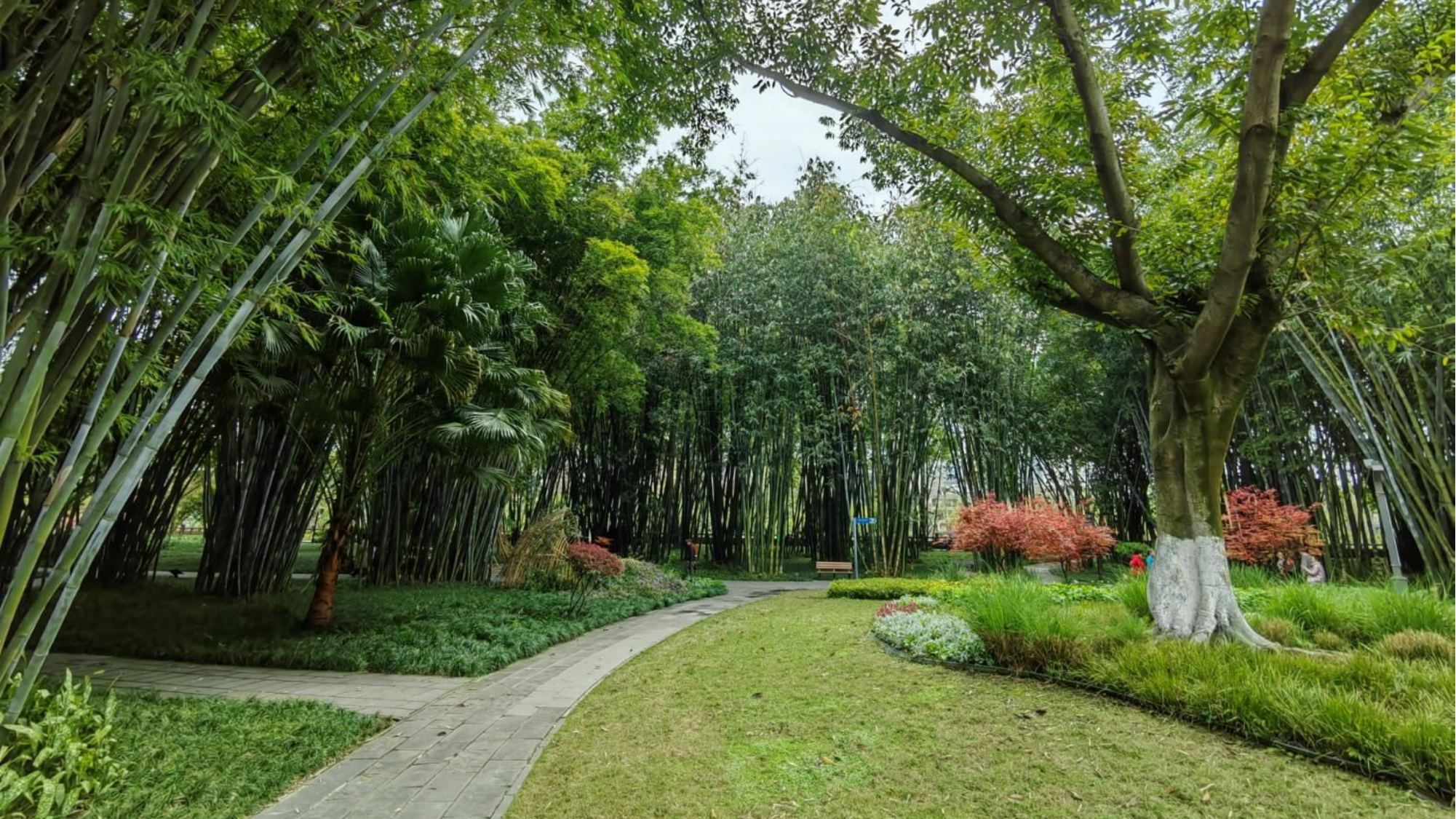

## Slide 23
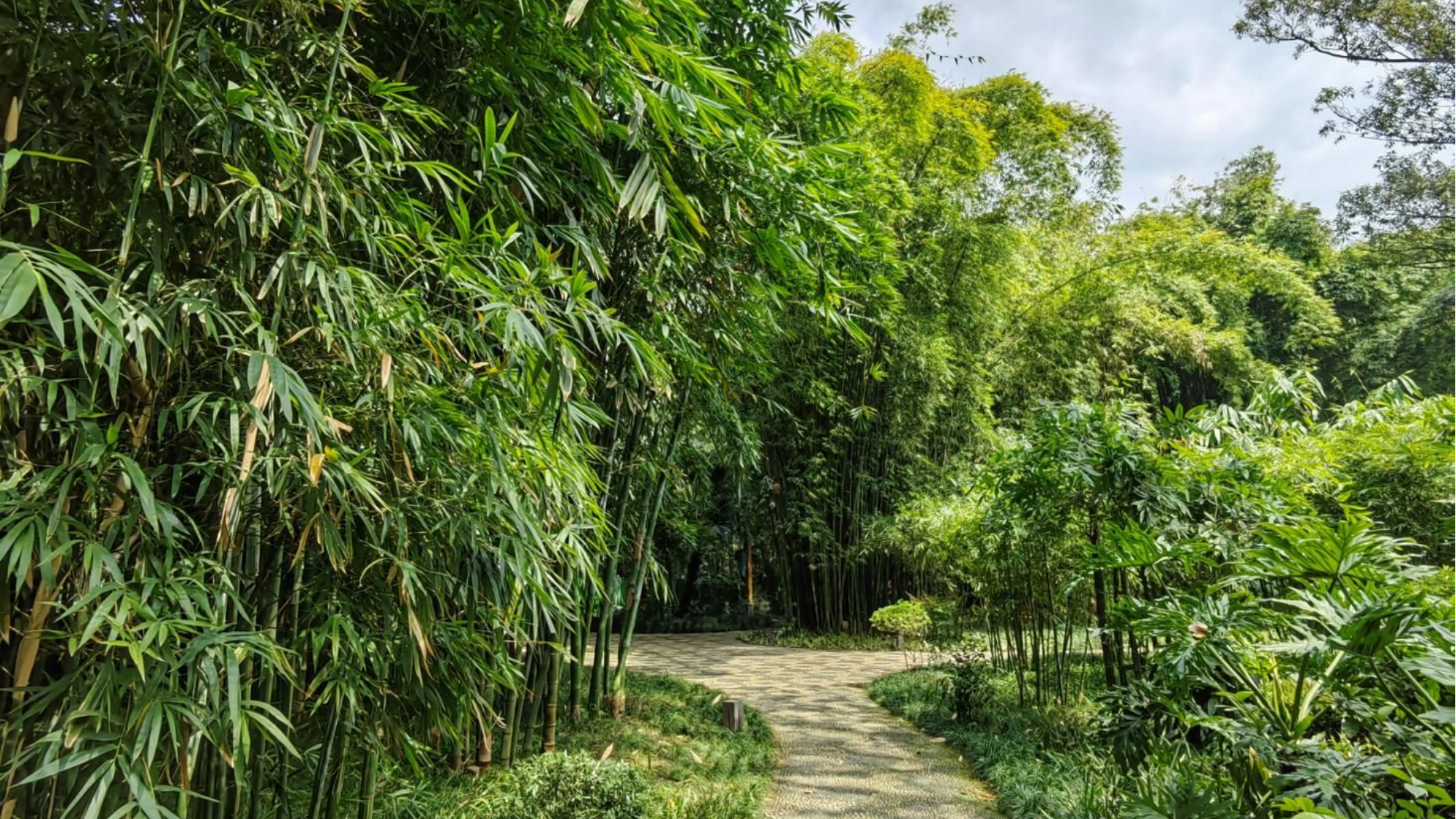

## Slide 24
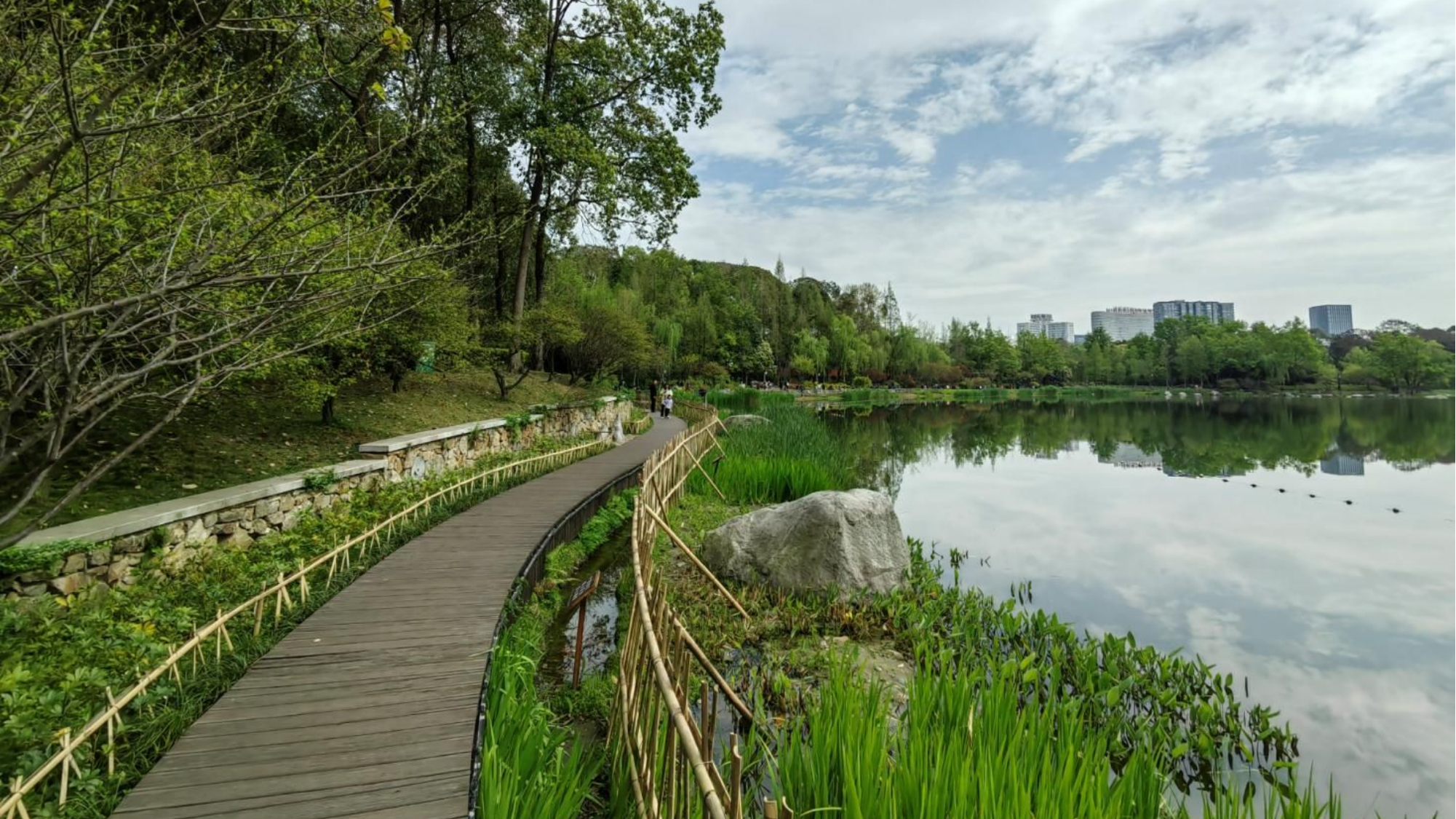

## Slide 25
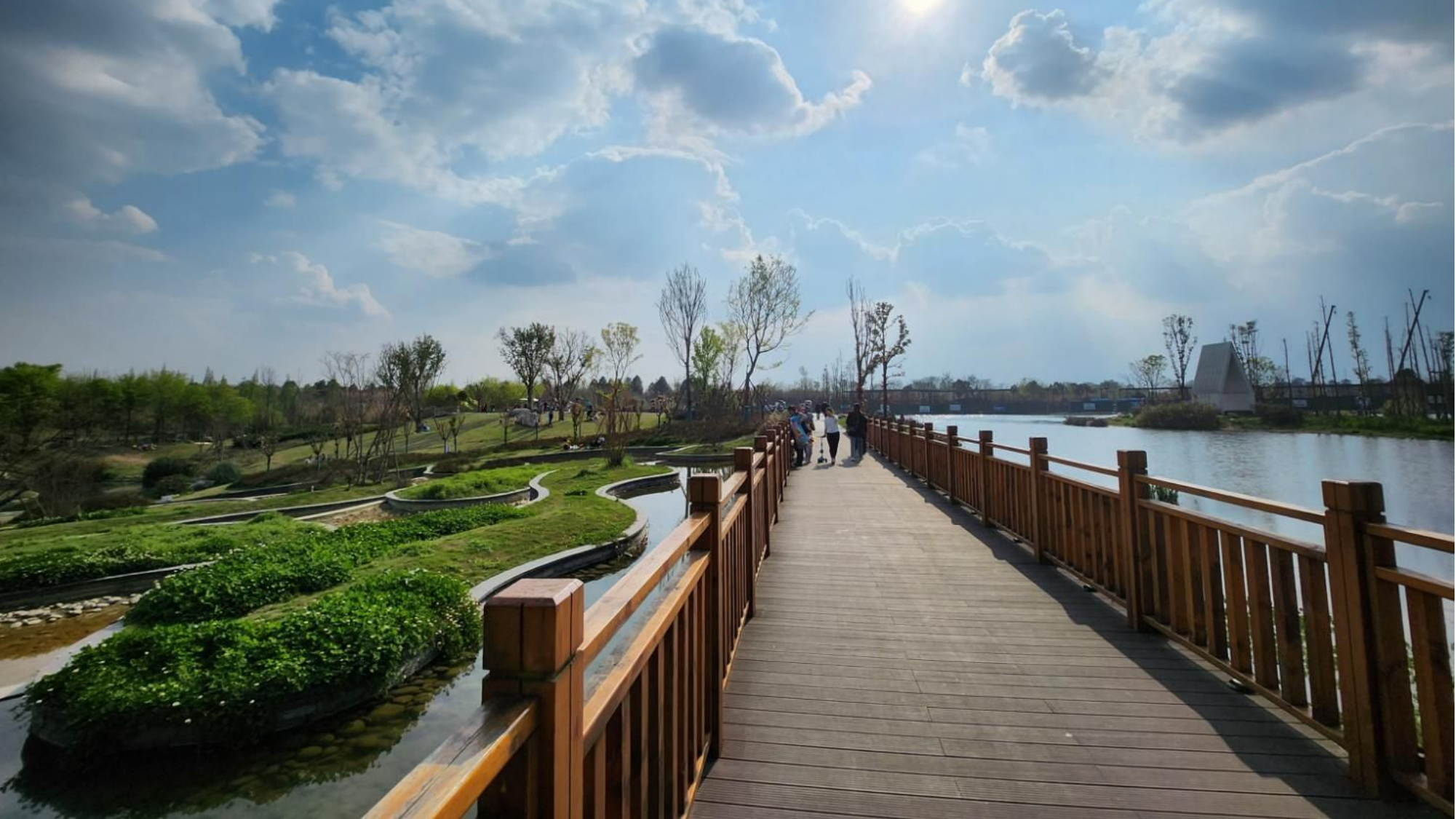

## Slide 26
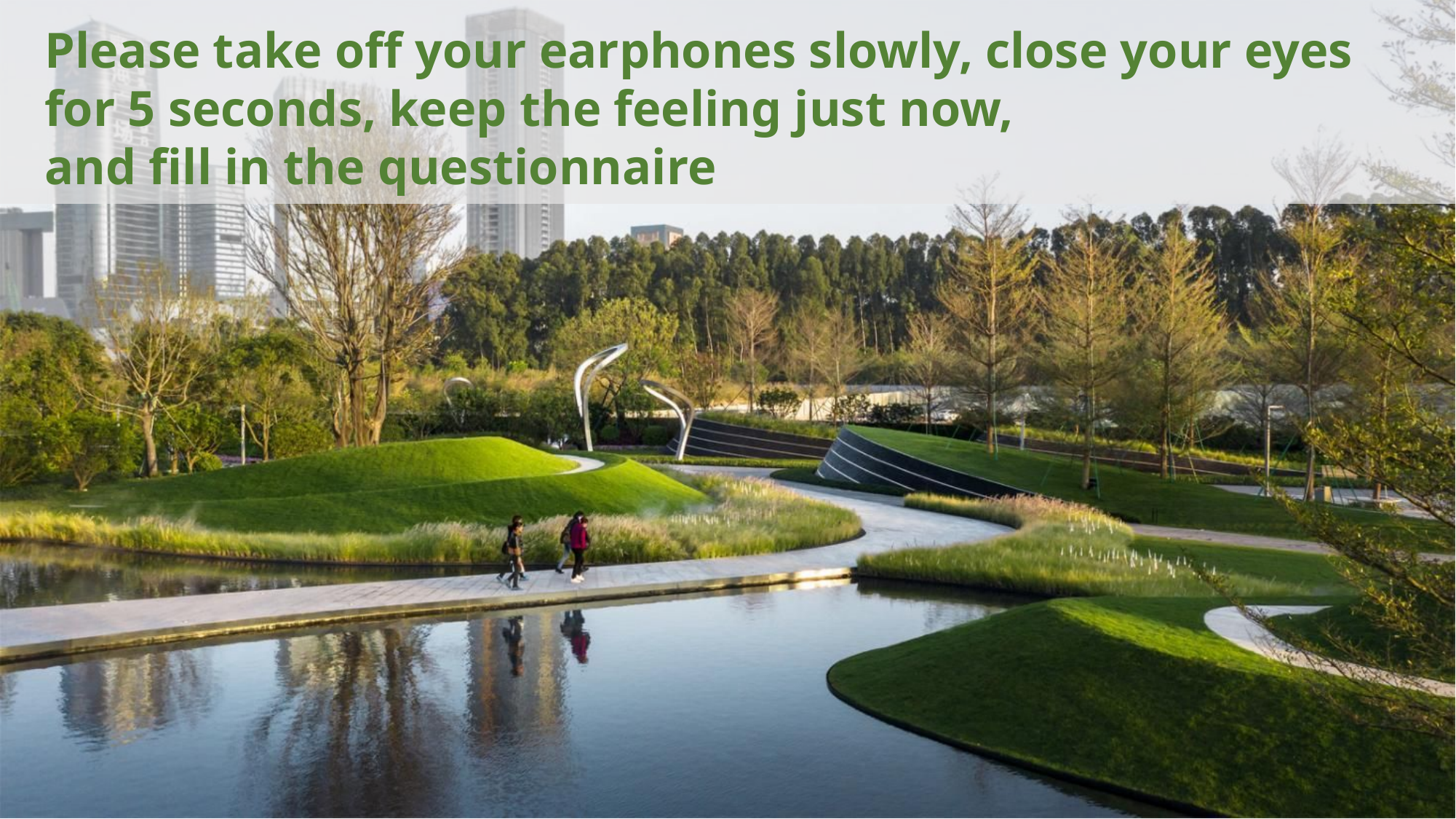

Please take off your earphones slowly, close your eyes for 5 seconds, keep the feeling just now,
and fill in the questionnaire
